# Morphological, biological, and genomic characterization of *Klebsiella pneumoniae* phage vB_Kpn_ZC2

**DOI:** 10.1186/s12985-023-02034-x

**Published:** 2023-05-03

**Authors:** Mohamed S. Fayez, Toka A. Hakim, Bishoy Maher Zaki, Salsabil Makky, Mohamed Abdelmoteleb, Kareem Essam, Anan Safwat, Abdallah S. Abdelsattar, Ayman El-Shibiny

**Affiliations:** 1 Center for Microbiology and Phage Therapy, Zewail City of Science and Technology, Giza, 12578 Egypt; 2 Microbiology and Immunology Department, Faculty of Pharmacy, October University for Modern Sciences and Arts (MSA), Giza, 11787 Egypt; 3 Department of Botany, Faculty of Science, Mansoura University, Mansoura, 35516 Egypt; 4 Faculty of Environmental Agricultural Sciences, Arish University, Arish, 45511 Egypt

**Keywords:** Gram negative, *Klebsiella pneumoniae*, Siphovirus, Multi-drug resistance (MDR), Bacteriophage, Phage therapy

## Abstract

**Background:**

Bacteriophages (phages) are one of the most promising alternatives to traditional antibiotic therapies, especially against multidrug-resistant bacteria. *Klebsiella pneumoniae* is considered to be an opportunistic pathogen that can cause life-threatening infections. Thus, this study aims at the characterization of a novel isolated phage vB_Kpn_ZC2 (ZCKP2, for short).

**Methods:**

The phage ZCKP2 was isolated from sewage water by using the clinical isolate KP/08 as a host strain. The isolated bacteriophage was purified and amplified, followed by testing of its molecular weight using Pulse-Field Gel Electrophoresis (PFGE), transmission electron microscopy, antibacterial activity against a panel of other *Klebsiella pneumoniae* hosts, stability studies, and whole genome sequencing.

**Results:**

Phage ZCKP2 belongs morphologically to siphoviruses as indicated from the Transmission Electron Microscopy microgram. The Pulsed Field Gel Electrophoresis and the phage sequencing estimated the phage genome size of 48.2 kbp. Moreover, the absence of lysogeny-related genes, antibiotic resistance genes, and virulence genes in the annotated genome suggests that phage ZCKP2 is safe for therapeutic use. Genome-based taxonomic analysis indicates that phage ZCKP2 represents a new family that has not been formally rated yet. In addition, phage ZCKP2 preserved high stability at different temperatures and pH values (-20 − 70 °C and pH 4 – 9). For the antibacterial activity, phage ZCKP2 maintained consistent clear zones on KP/08 bacteria along with other hosts, in addition to effective bacterial killing over time at different MOIs (0.1, 1, and 10). Also, the genome annotation predicted antibacterial lytic enzymes. Furthermore, the topology of class II holins was predicted in some putative proteins with dual transmembrane domains that contribute significantly to antibacterial activity. Phage ZCKP2 characterization demonstrates safety and efficiency against multidrug-resistant *K. pneumoniae*, hence ZCKP2 is a good candidate for further in vivo and phage therapy clinical applications.

**Supplementary Information:**

The online version contains supplementary material available at 10.1186/s12985-023-02034-x.

## Introduction

Antibiotic resistance is increasing in all parts of the world, posing a serious threat to public health management practices [1]. Antibiotic-resistant strains were only seen in hospitals, but they are now prevalent throughout the world [2]. It is estimated that by 2050 if no new drug is discovered, there will be no effective antibiotics available [3]. Numerous infections have high rates of morbidity, death, and financial expense. One of the most significant public health concerns of the twenty-first century is antimicrobial resistance (AMR), which threatens the effective prevention and treatment of an increasing number of bacterial infections [4]. Resistance to antimicrobials occurs as pathogenic bacteria degrade antibacterial medicines, change their proteins, and alter their membrane permeability to antibiotics [5]. Bacteria that cause common to serious infections have developed resistance to the majority of available antibiotics on the market to varying degrees over several decades [6]. Our capacity to treat common diseases will decrease due to the emergence and spread of drug-resistant bacteria with new resistance mechanisms [7].

The most serious and commonly occurring Gram negative infections that occur in health care settings are caused by Enterobacteriaceae (mostly *Klebsiella pneumoniae*), *Salmonella*, and *Escherichia coli* [8, 9]. Gram negative *Klebsiella pneumoniae* is one of the most common Gram negative pathogens associated with a wide spectrum of community and hospital-acquired infections, such as urinary tract infection (UTI), pneumonia, intra-abdominal infection, bloodstream infection (BSI), meningitis, and pyogenic liver abscess (PLA) [10]. Globally, the prevalence of extended-spectrum cephalosporin-resistant *K. pneumoniae* producing extended-spectrum -lactamases (ESBL) has risen dramatically in recent decades [11]. ESBLs hydrolyze the beta lactam ring thereby inactivating the antimicrobial compounds such as penicillin and cephalosporin. The third generation of cephalosporins can hinder the treatment because ESBLs can suppress the oxyimino cephalosporins. [12]. As per Centers for Disease Control and Prevention (CDC) report in 2019, 80% of the 9000 infections that have been reported by carbapenem-resistant Enterobacteriaceae (CRE) in 2013 were caused by AMR *K. pneumoniae* [13]. CRE are among the top level of the WHO list of antibiotic-resistant “priority pathogens” that pose the greatest threat to human health [14].

A new approach to eradicating the spread of antibiotic-resistant bacteria is required. With the growing concern over antibiotic resistance, interest in phage therapy as a possible solution to the problem that is expanding rapidly. Phage therapy is being used to control pathogenic bacterial infections especially multiple antibiotic-resistant bacterial infections and as potential anti-inflammatory and immunomodulatory agent [15]. Their therapeutic potential in medicine to control MDR pathogens is due to their specificity and potency in inducing lethal effects in the host bacterium by cell lysis [16]. In the context of therapeutics, only virulent phages can be used. Strictly virulent phages can attack particular bacterial strains and contribute to lytic infection associated with metabolic disturbance and cell lysis, which decreases the number of bacterial cells found in the infected human host to a level that presents no danger or harm to the organism [17].

Globally, *K. pneumoniae* phages have been isolated from various sources, including sewage [18], ponds [19], rivers [20], seas [21], farm wastewater [22], water troughs [23], animal feces [24], and clinical samples [25, 26], with sewage from hospitals being the most common source [27–33]. The majority of the isolated phages of *K. pneumoniae* are members of the class *Caudoviricetes*, which is characterized by being naked (non-enveloped), dsDNA and tailed [34]. Nonetheless, a *K. pneumoniae* tailless phage with a tectivirus morphotype was also isolated [19]. In addition, many of the isolated *K. pneumoniae* phages demonstrated anti-capsule and anti-biofilm activity by expressing various types of polysaccharide depolymerases [35, 36, 39].

Prior to the genomic era, the only way to ensure the safety and taxonomy of isolated phages was to use phenotypic assays, which are nearly impossible to reveal everything encoded in the genome [37]. Since DNA sequencing methods have advanced over the last two decades, most laboratories can now sequence the genomes of bacteriophages [38]. Phages for phage therapy can be checked for any unfavorable encoding genes, such as lysogeny-encoding genes and virulence or resistance genes that may be transmitted to bacteria; therefore, genomic analysis and bioinformatics could reduce the efforts required for a safety study [39].

The effectiveness of such a strategy is dependent on two variables: the consistency with which phage resistance develops in vitro and the degree to which the resistance produced in vitro coincides with the resistance produced in vivo [40]. This paper discusses the isolation and characterization of an effective vB_Kpn_ZC2 (ZCKP2) phage against MDR-KP.

## Materials and methods

### Bacterial growth condition

Thirty sputum-clinical isolates of MDR *Klebsiella pneumoniae* were employed in this study. The bacterial isolates were collected from the lab stock at Zewail City of Science and Technology. Twenty isolates (KP/01 - KP/20) were previously characterized by Fayez et al. [41], while the remaining ten *Klebsiella* isolates (K1, K6, K7, K/10, K18, K/20, K24, K25, K/30, and K31) were previously characterized by Zaki *et al*. [39]. Fresh bacterial cultures were made before each experiment by inoculating one colony from MacConkey agar (Oxoid, England) into 1 mL of TSB in 1.5 mL centrifuge tube and incubated for 16 h at 37°C with shaking (200 rpm).

### 16S rRNA gene sequencing

PCR amplification and sequencing were performed to confirm the identity of the *K. pneumoniae* isolate (KP/08) using specific and universal primers for the 16s rRNA gene forward primer (5’-AGAGTTTGATCCTGGCTCAG-3’), and the reverse primer (5’-TACGGYTACCTTGTTACGACTT-3’). Thirty cycles were performed at a denaturation temperature of 94°C for 30 s; annealing at 55°C for 30 s and extension at 72°C for 1 min. The PCR product was run on a 1.5% (w/v) agarose gel to identify its size [42]. Following the manufacturer’s instructions, the amplified 16S rRNA gene fragment was purified using QIAEX II Gel Extraction Kit (QIAGEN, Hilden, Germany) [43]. Finch TV software (https://digitalworldbiology.com/FinchTV/) was used to process the 16S rRNA gene’s acquired nucleotide sequence. The isolated strain was identified using BLASTn (accessed on 9 SEP 2022, at Basic Local Alignment Search Tool, https://blast.ncbi.nlm.nih.gov/Blast.cgi,) against the 16S ribosomal RNA database [44]. The sequence was deposited in the NCBI GenBank database under accession number OP410967.1

### Isolation, purification, and amplification of bacteriophage

Six different phages were obtained from sewage water in Giza, Egypt. The water samples were centrifuged at 4000 rpm and the supernatant was filtered from other bacteria using 0.2 µm porous syringe filters [45]. Using enrichment techniques for phage isolation, 10 mL of sewage samples were combined with 1 mL of an overnight culture from KP/08, incubated at 37°C for 4h, then mixed with 1% chloroform and centrifuged at 5000 rpm for 20 minutes while the supernatant was kept. Following this, a spot assay was performed using a mixture of 100 µl of the bacterial host culture and 4 mL of soft agar (0.5% w/v agar) then poured into a TSA plate. From each supernatant 10 µL aliquots were spotted in triplicate on bacterial lawns, and the plates were then incubated at 37°C for 24 h. Phage clear plaques were purified with repeated isolation of a single plaque using sterile micropipette tips. All isolated phages were amplified in liquid culture (TSB), and the lysates were centrifuged at 5,000 g at 4°C for 15 min [46, 47]. Then the supernatant containing phages was centrifuged for 1 h at 15,300 ×g at 4 °C. The pellet was resuspended in SM buffer (100 mM MgSO4.7 H2O; 10 mM NaCl; 50 mM Tris-HCl; pH 7.5) and purified through 0.22 µm syringe filters (Chromtech, Taiwan) 26]. Bacteriophage titers were determined using double agar overlay plaque assays and spotted in triplicate onto bacterial lawns [48]. The isolated phages were enriched and propagated in TSB, 100 mL of host was infected with each phage separately and incubated at 37°C with 120 rpm shaking to increase phage stocks [49].

### Phage characterization

#### Pulsed field gel elecrophorises

For Pulsed Field Gel Electrophoresis (PFGE), DNA was prepared from bacteriophage vB_Kpn_ZC2 (ZCKP2) (10^10^ PFU/mL) to determine the genome size [50]. Fist, the bacteriophage suspended in agarose plugs were digested with lysis buffer (0.2% w/v SDS [Sigma, Gillingham, UK]; 1% w/ N-Lauryl sarcosine [Sigma, Gillingham, UK]; 100 mM EDTA; 1 mg/mL Proteinase K [Fischer Scientific]), then left overnight at 55 °C. After being washed with a washing buffer, two slices of agarose-containing DNA were placed into the wells that contain 1% w /v agarose gel. By using a Bio-Rad CHEF DRII system, the gel was run in 0.5 X Tris-borate-EDTA, at 200 V at 14 °C for 18 h with a switch time of 30 to 60 s. The genome’s size was calculated by comparing using standard concatenated lambda DNA markers of range 48.5–1,018 kb (Sigma Aldrich, Gillingham, UK).

#### Examination of phage by Transmission Electron Microscopy (TEM)

The morphology of phage ZCKP2 was examined by glow-discharged (1 min under vacuum) by using TEM at the National Research Center (Cairo, Egypt) [51, 52]. Formvar carbon-coated copper grids (Pelco International) were submerged into phage suspension. (2.5% v/v) glutaraldehyde was used to fix the phage, which was rinsed, and stained using 2% phosphotungstic acid (pH 7.0). After drying, grids were examined using a high-resolutiontransmission electron microscope (JEOL 1230).

#### Phage host range

The host range of phage ZCKP2 was determined against 30 clinical isolates of MDR *K*. *pneumoniae* by using the spot assay in triplicate as previously mentioned [48, 53]. Briefly, 100 µl of freshly prepared culture of each bacterial strain was added to 4 mL of 0.5% top agar, which was subsequently poured onto a base TSA agar. Ten microliters of phage lysate at a titer of 10^10^ PFU/mL were spotted onto freshly lawns of the bacterial strains and incubated overnight at 37 °C.

#### Relative efficiency of plating

The relative efficiency of plating (EOP) of the phage ZCKP2 was conducted by counting plaques of clear lysis after plating 10-fold serial dilutions of the phage onto fresh lawns of each susceptible bacterial strain. The plaque enumeration was accomplished by using the spotting assay over a double agar overlay. Then, EOP was calculated by dividing the enumerated PFU on each bacterial strain by the counted PFU on the isolating host. Subsequently, the EOP was classified into high (≥ 0.5), medium (0.5 − 0.1), or low (0.1 – 0.001) [54].

#### One-step growth curve

The eclipse period, latent period, lysis time and burst size of the ZCKP2 phage were defined by observing dynamic variations in the number of phage particles throughout a replicative cycle (modified from) [55]. Shortly, host strain KP/08 was grown at 37 °C to exponential phase (~ 10^8^ CFU/mL) and incubated with ZCKP2 phage (~ 10^7^ PFU/mL) at an MOI of 0.1. Aliquots of the infected culture were serially diluted at each time point to count the number of phages present using a spotting test [53]. Two aliquots were collected at each time point, the first one was treated with 1% (v/v) chloroform to induce the release of the intracellular phage, while the second one was spotted without chloroform to determine the phage infective center (IC). The ratio of the released phage titer to the original number of infected cells was used to calculate the burst size per infected cell. By deducting the average of the early time point phage titers treated with chloroform (before the end of the eclipse period) from that of the IC early titers, the number of infected cells was calculated [56]. The experiment was conducted in triplicate.

#### Time-killing curves

The bacterial killing activity was determined for the phage ZCKP2. The experiment lasted for 325 min, using ZCKP2 phage at different MOIs (0.1, 1, 10, and 100) against strain KP/08 at 37 °C to exponential phase (~ 10^7^ CFU/mL) [53]. Throughout the time, the optical density (O.D 600 nm) was measured using 96-well plate (FLUOstar Omega, BMG LABTECH, Ortenberg, Germany). Data were collected at 25 min intervals for around 5.5 h using the MARS Data Analysis Software package (version 3.42). The bacterial culture without phage inoculation was used as a control. The experiments were performed in triplicate.

#### Phage temperature and pH stability

The stability of bacteriophages over a range of temperatures was evaluated by incubating 20 µl of the phage suspensions in 180 µl SM buffer at -20, 4, 40, 50, 60, 70, 75, and 80 °C for 4 h. The bacteriophage samples were taken after 4 h. of incubation to detect the change in phage titer upon sudden temperature alteration [49]. The titers of released bacteriophages were determined using serial dilutions and spotting using double agar overlay plaque assays. By incubating 10 µl of bacteriophage suspensions in 990 µl of TSB at different pH values (2, 3, 4, 5, 7, 9, 11, 12, and 13), and the viability of the bacteriophage was determined over time. After 4 h of incubation, pH samples were taken to determine the phage titer using serial dilutions and spotting as described before to evaluate the titers of released bacteriophages [53].

#### Phage genome sequancing and characterization

Genomic DNA was extracted from phage ZCKP2 (10^10^ PFU/mL) lysates using proteinase K (100 g/mL in 10 mM EDTA pH 8), and then resin purification using the Wizard DNA kit (Promega, UK) in accordance with the manufacturer’s instructions. DNA sequencing was carried out using the Illumina MiSeq platform. Then the Illumina Nextera tagmentation protocol (Illumina, Cambridge, UK) was used for library preparation. The data included 150 bp length paired-end sequences. FASTQC was used to access the sequences’ precision [57]. Using SPAdes [58] and K-mers of 21, 33, 55, 77, and 99, sequences were de novo assembled, producing a 48.2 kbp unique contig.

BLASTn was performed against the nucleotide collection database to find the closely matching phages After that, MEGA-X ages were imported with the best-matched phages [59] to draw a phylogenetic tree using the CLUSTAL-W aligner [60] and the best Maximum Likelihood fit model (GTR: General Time Reversible substitution model, G: Gamma distributed among sites). The NCBI ORF finder search server was used to identify open-reading frames using methionine and alternate initiation codons as the start codon. The putative coding sequences were then identified by comparing the predicted ORFs to the NCBI non-redundant protein sequences (nr) database using BLASTp (CDSs) with considering an e-value cutoff < 10^− 7^. Moreover, the predicted ORFs and coding sequences were matched to those predicted by PHASTER [61]. An additional round of ORFs prediction and functional annotation was performed on RASTtk [62–64] and BV-BRC [65] to increase confidence in the predicted encoding gene.

SnapGene Viewer (GSL Biotech; available at https://www.snapgene.com/; access on 9 SEP 2022) was used to create the circular genomic map. Phage ZCKP2’s annotated whole genome was uploaded to the GenBank database with the accession number NC_071151. Phage ZCKP2 suitability for therapeutic application was assessed using PhageLeads, which checked the genome for temperate markers, antibiotic resistance genes, and virulence genes [66]. DeepTMHMM was used to analyse the putative proteins and detect transmembrane topology [67].

The phylogenetic analysis of the phage ZCKP2 was conducted by different approaches. The phages of high similarity to phage ZCKP2 were identified by BLASTn, and their pairwise intergenomic similarities were computed by the Virus Intergenomic Distance Calculator (VIRDIC), in which the default thresholds for species (> 95%) and genus (> 70%) were applied [68]. Viral Proteomic Tree (ViPTree) was used to generate a proteomic tree of the ZCKP2 genome based on genome-wide sequence similarities computed by tBLASTx [69]. Additionally, the orthologous (signature) genes of the closely related phages were predicted by using CoreGenes 0.5 [70]. Accordingly, conserved phage proteins were used to perform phylogenetic analysis based on the alignment of their amino acid sequences. The conserved proteins were aligned using CLUSTAL-W and analysed by the best Maximum Likelihood fit model in MEGA 11 [71, 72].

### Statisical analysis

All experiments were conducted in triplicate, and the results were illustrated in the form of mean ± standard deviation (SD). In this study, GraphPad Prism 9.1.1 software was used to generate graphs and perform all statistical analyses. Both Student’s *t*-test (two-tailed) and ANOVA tests were used during the work to evaluate the significance of *p* < 0.05.

## Results

### 16 S rRNA gene sequence

The 16S rRNA sequence for the main bacterial host KP/08 was performed afterward (GenBank Acc. No. OP410967.1). BLASTn of the 16S rRNA sequence had a 99% sequence identity to the *K. pneumoniae* strain.

### Morphology of vB_Kpn_ZC2 phage

**Fig. 1:**
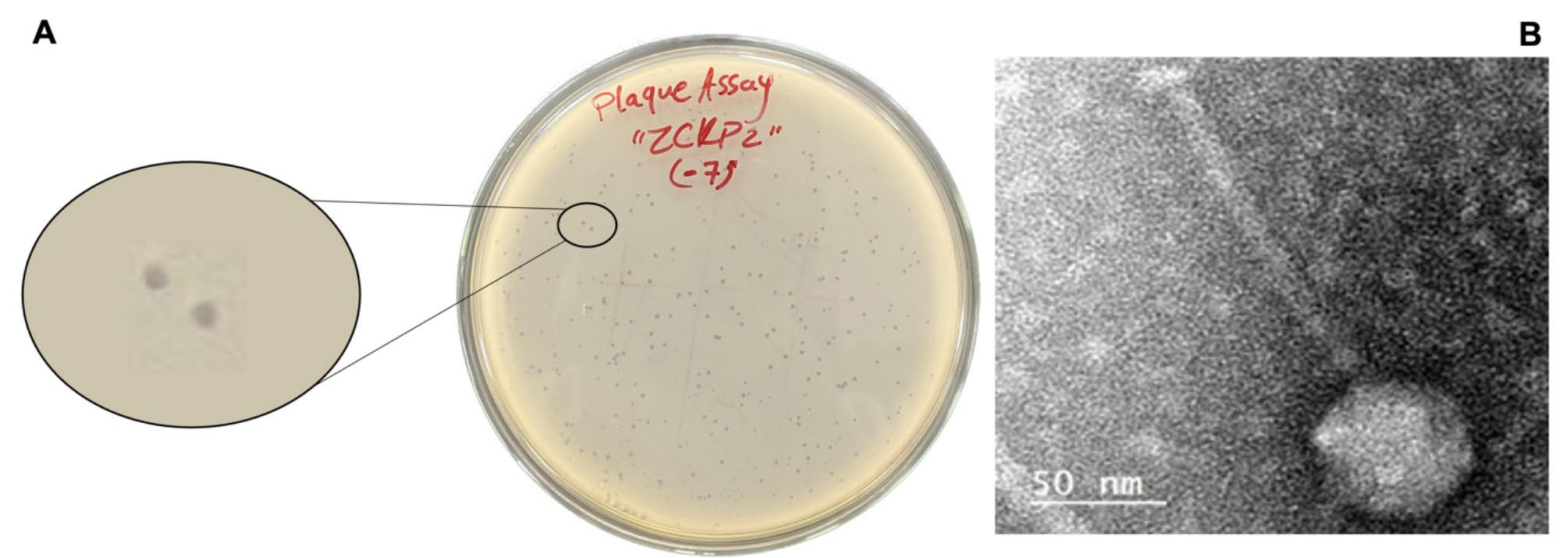
Visualization of the isolated phage ZCKP2. **A**. Clear plaques of similar morphology for ZCKP2 over double-layer agar. **B**. Transmission electron microscopic image of phage ZCKP2.

Isolated phage was initially screened against the *K. pneumoniae* strain through a spot test. A clear zone over a bacterial lawn was observed due to the lytic activity of the phage. On double-layer agar plates, vB_Kpn_ZC2 (ZCKP2) phage produced small but clear plaques of similar morphology (Fig. 1A). TEM micrographs showed phage ZCKP2 with an icosahedral head, a filamentous, cross-banded, and non-contractile tail; these morphological findings are characteristics of siphovirus (Fig. 1B). The phage proportions were measured on virions and the head diameter is ~ 65 nm, and the tail length is ~ 160 nm.

### Bacteriophage host range and relative efficiency of plating

Seven bacterial isolates, out of 30 screened isolates, were susceptible to the phage ZCKP2 which produced clear lysis zones on them. The phage ZCKP2 was observed against the susceptible isolates of *K. p*neumoniae in terms of relative EOP as in Table 1.

**Table 1:** EOP for phage ZCKP2 against *K. pneumoniae* isolates

Bacterial Species	EOP
EOP ≥ 1	4
EOP < 1	3

### One step growth curve

**Fig. 2:**
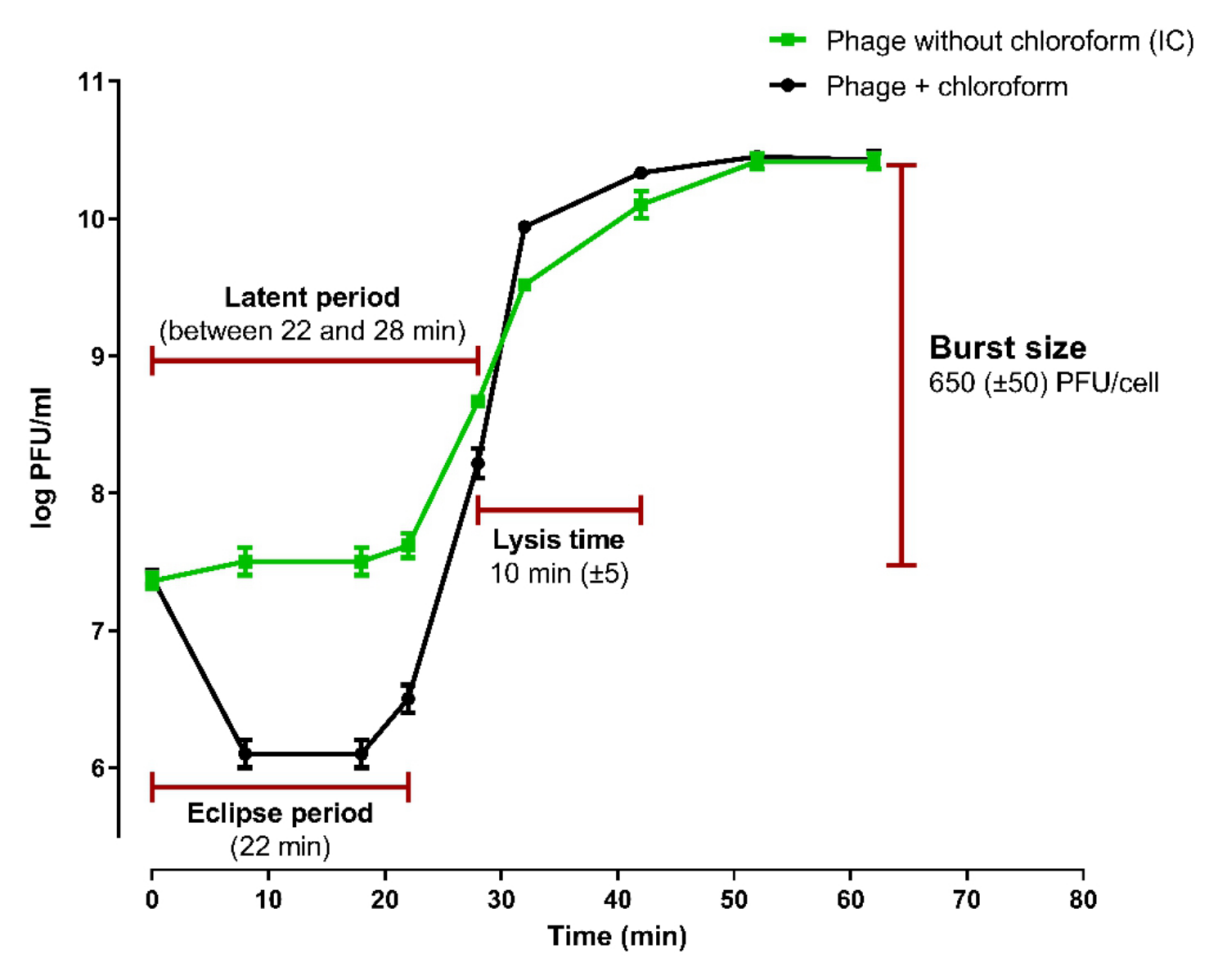
One-step growth curve of phage ZCKP2 at MOI 0.1. The titers phage ZCKP2 was enumerated by spotting assay. Data points represent the mean of PFU/mL at different intervals during the experiment time

The observed phage replication kinetics in the one-step growth experiment revealed that the ZCKP2 phage had about 22 min eclipse period and a latent period of 25 (± 3) min, followed by 10 min lysis. ZCKP2 phage demonstrated a large burst size of about 650 (± 50) PFU/mL per infected bacterial cell (Fig. 2).

### Time-killing curves

**Fig. 3:**
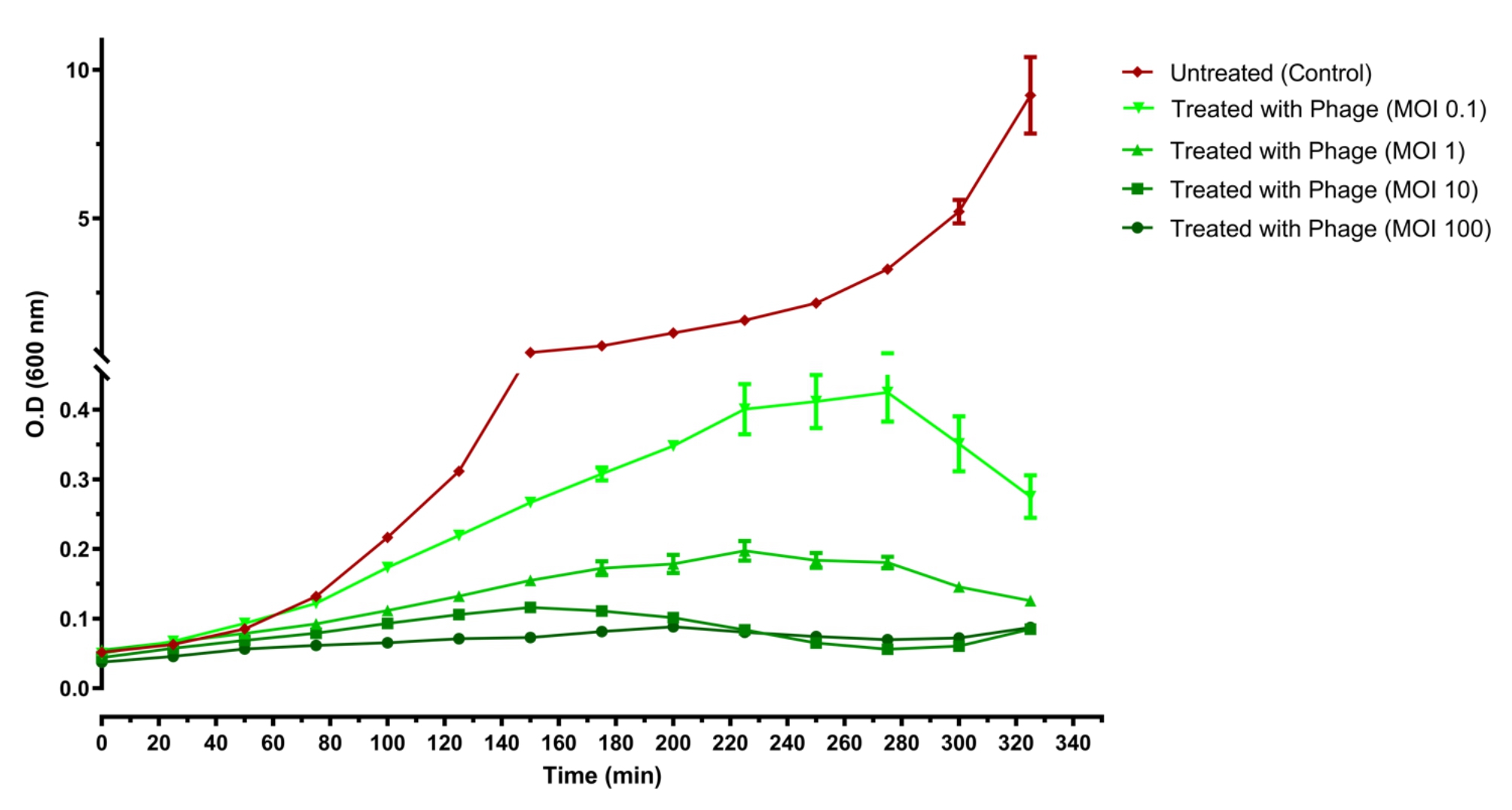
Time-killing Curve of *Klebsiella* strain KP/08 using ZCKP2 phage at different MOIs (0.1, 1, 10 and 100) over 325 min in a shaking condition.

The time-killing curves displayed a reduction in the optical density O.D at 600 nm for the groups treated with phage, unlike the untreated group. Moreover, the bacteria treated with higher MOIs (10 and 100) presented a faster reduction in the O.D readings (Fig. 3). After the 325 min, the untreated bacteria were at the O.D of 10.8 ± 0.96; at MOI 0.1, the O.D was 0.275 ± 0.05, at MOI 1, the O.D was 0.125 ± 0.01; at MOI 10, the O.D was 0.085 ± 0.01; and at MOI 100, the O.D was 0.095 ± 0.01. At higher MOIs (10 and 100), the bacteria started to display increase in the O.D reading, which might reflect the probable phage resistance.

### Phage temperature and pH stability

**Fig. 4:**
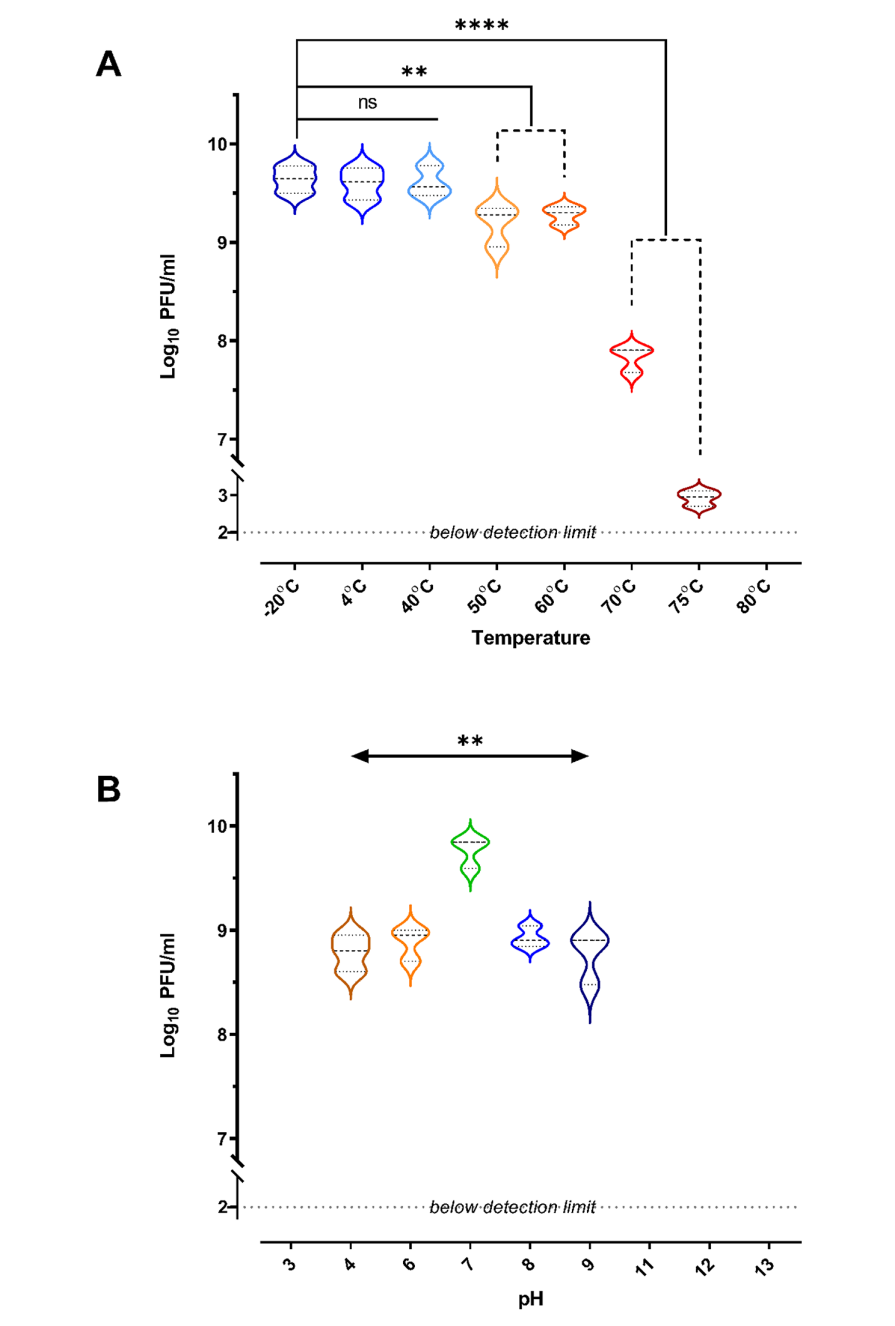
Violin plots of the thermal and pH stability of phage ZCKP2. The phage stability is represented as enumerated titer (log_10_ of PFU/mL). **A.** Thermal stability: phage titer at -20 °C was compared to other temperatures’ phage titers. **B**. pH stability: phage titer at pH 7.0 was compared to phage titers at the other pH values. The mean of phage titer triplicates was calculated, and statistically analyzed by t-test at a significance level of P < 0.05.

The phage ZCKP2 demonstrated high stability at storage temperature (-20 and 4°C) which was comparable to its stability at incubation temperature (40 °C) (Fig. 4A). Regarding thermal stability (above 40 °C), the phage was stable with a slight reduction in its titer at range of 50–60 °C. The phage dramatically reduced at temperature above 70 °C that even became undetectable at 80 °C.

The most optimum pH was 7.0, in addition, the phage ZCKP2 demonstrated acceptable stability with limited titer reduction at range of pH 4.0 to 9 (Fig. 4B). However, the phage did not tolerate the higher acidity (≤ pH 3.0) and alkalinity (≥ pH 11.0) conditions since no phage titers were determined.

### Genome annotation and bioinformatics analysis

**Fig. 5:**
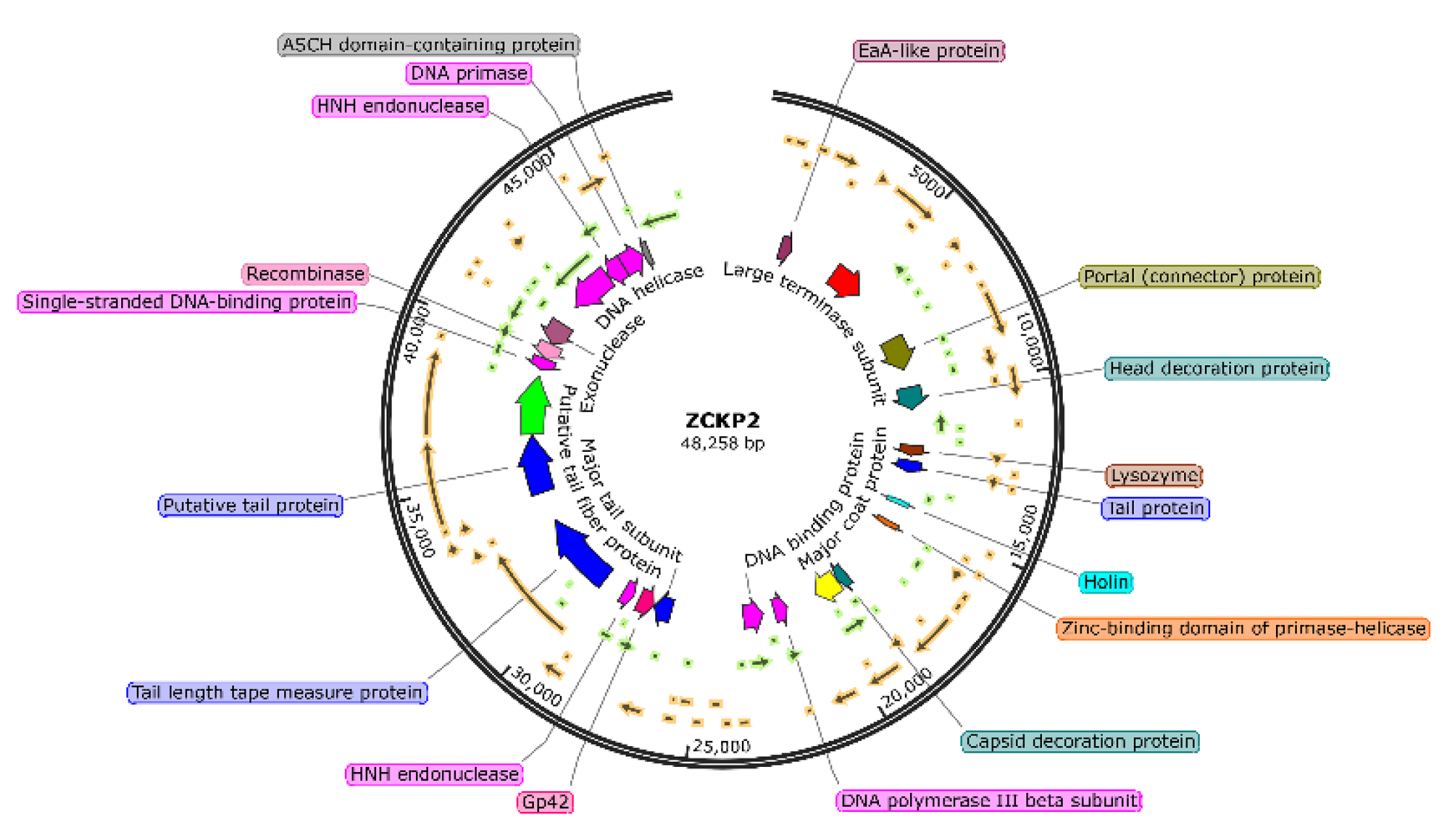
A genomic map of phage ZCKP2, and the predicted coding sequences with a signed functions are labelled on the genomic map.

Pulse Field Gel Electrophoresis (PFGE) estimated the size of the double-stranded DNA genome of the phage ZCKP2 at about 48.2 kbp. The phage ZCKP2’s whole genome was sequenced and added to the GenBank database (GenBank Acc. No. NC_071151). The sequenced reads of phage ZCKP2 were assembled as one contig of size 48,258 bp, a G + C content of 47.5%, and sixty-nine ORFs. The functional genes are highlighted on the genomic map (Fig. 5). Twenty-eight putative proteins have been ascribed roles, including DNA replication/transcription/repair proteins, DNA packaging proteins, structural proteins, and cell lysis proteins. The list of the putative protein-coding genes was manually curated and listed in Supplementary Table S1. Further genomic analysis uncovered one tRNA-Arg gene with the anticodon sequence TCT.

**Fig. 6:**
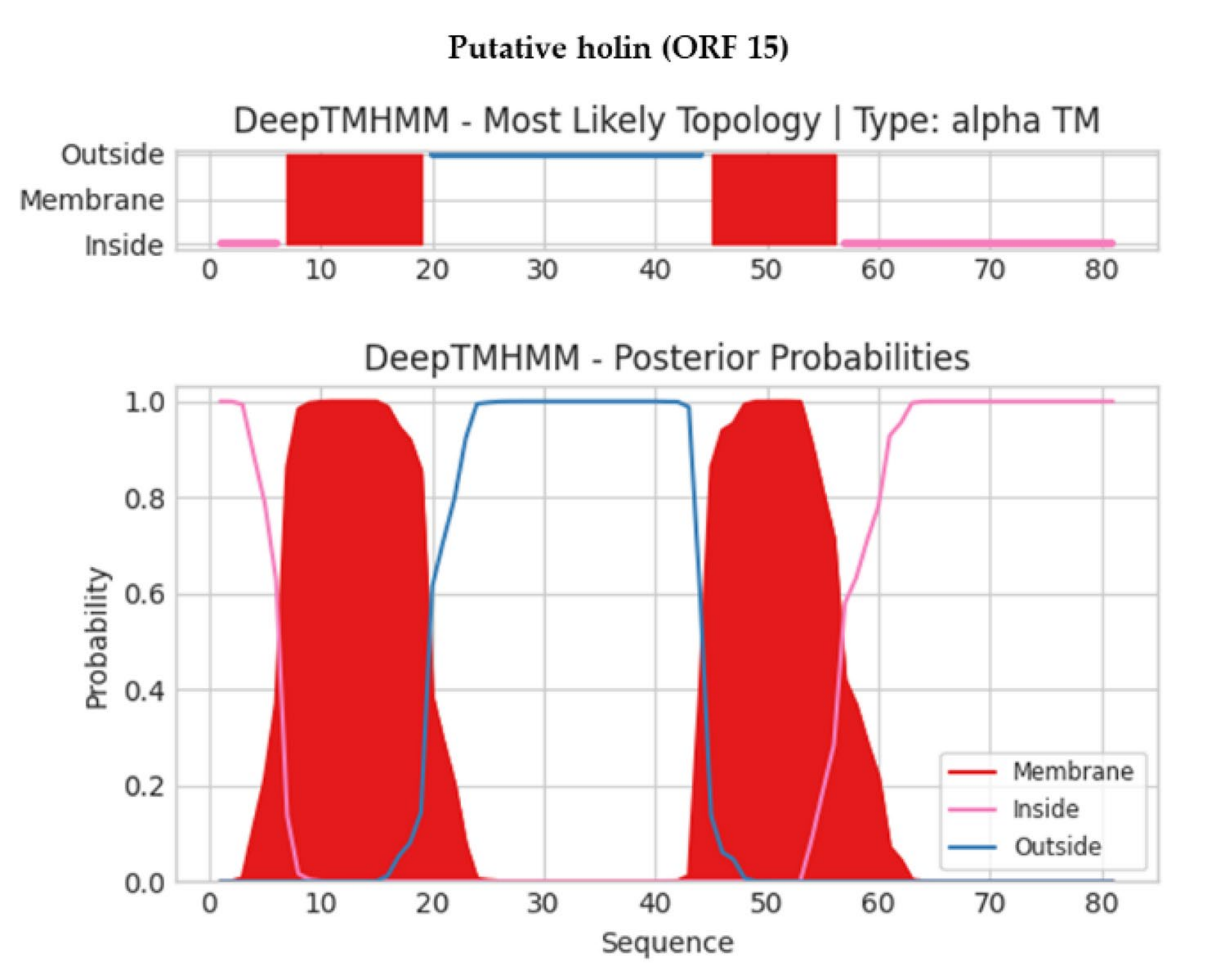
Predicted transmembrane topology using the DeepTMHMM tool of putative holin (ORF 15). Red blocks represent the predicted transmembrane domains, while the pink line and blue line represent the domains inside and outside the membrane, respectively. The Y-axis represents the prediction probability, while the X-axis represents the amino acids sequence position.

None of the predicted ORFs encodes any lysogenic phage-related proteins, such as transposases or integrases. Also, PhageLeads screened the phage genome, and no genes were predicted to have the potential for the temperate life cycle, antibiotic resistance, or bacterial virulence. The latter results indicate the safety and applicability of phage ZCKP2 for therapeutic purposes. The putative proteins were analyzed using DeepTMHMM for transmembrane domains (TMDs), which were predicted in five putative proteins (ORFs: 11, 15, 16, 28, and 35; Figure S1). The topology of two TMDs was detected in a putative holin (ORF 15), as described in Fig. 6.

### Phylogenetic analysis

**Fig. 7:**
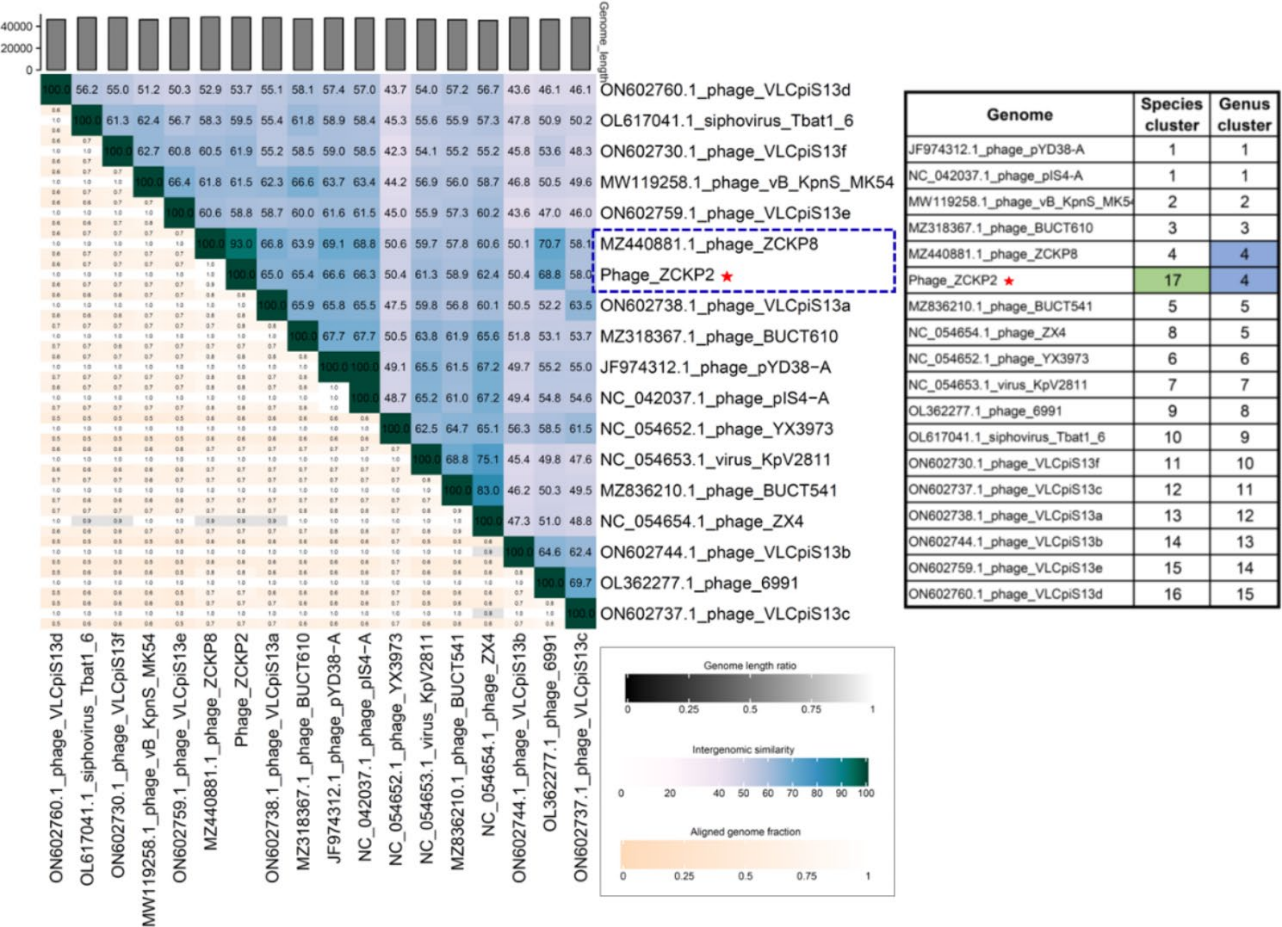
Virus Intergenomic Distance Calculator (VIRIDIC) heatmap for the intergenomic similarity between ZCKP2 and top-matched phages on BLASTn.

**Fig. 8:**
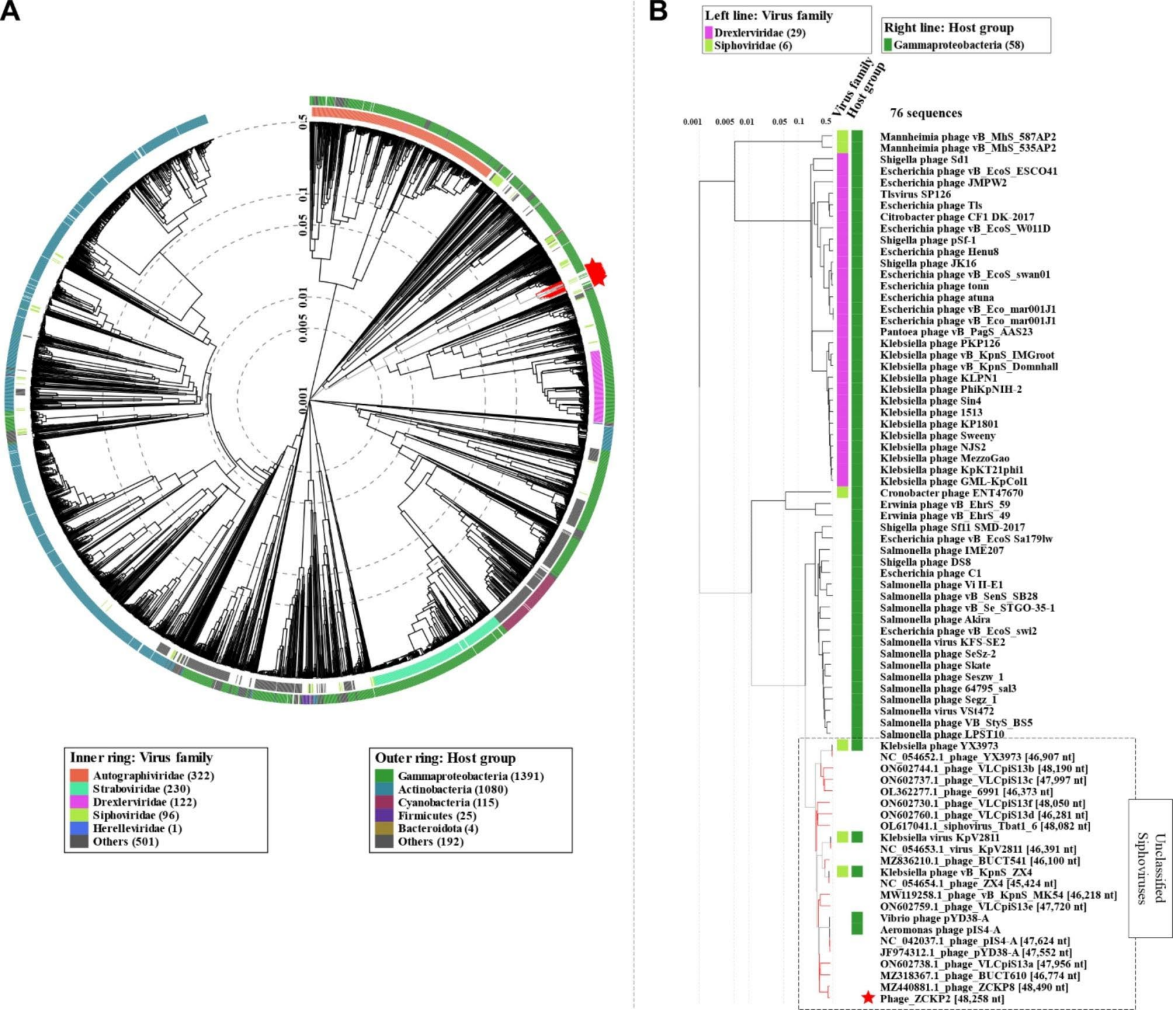
Proteomic tree generated by ViPTree of phage ZCKP2. **(A)** Circular proteomic tree of phage ZCKP2, top BLASTn hits, and related phages of RefSeq genomes. **(B)** Rectangular tree represents a subset of the closely related phages from circular tree.

**Fig. 9:**
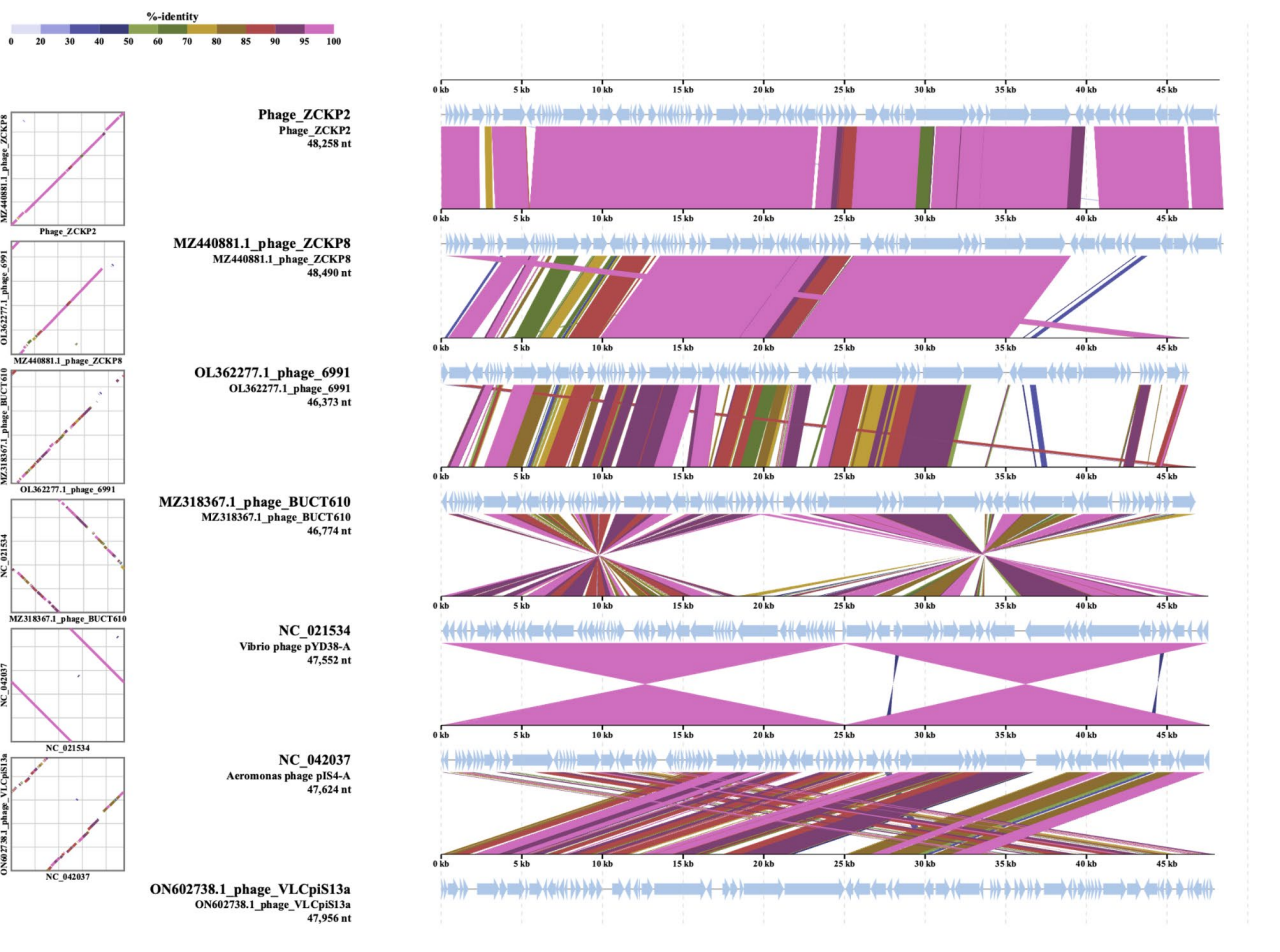
Whole-genome alignment and comparison between the phage ZCKP2 and closely related phages.

VIRDIC computed the intergenomic similarity of phage ZCKP2 and the top-matched phages, and only *Klebsiella* phage ZCKP8 was within the genus threshold (93% intergenomic similarity). Therefore, phage ZCKP2 and *Klebsiella* phage ZCKP8 were clustered into the same genus but different species (Fig. 7). Likewise, the proteomic tree inferred that phage ZCKP2 is closely related to *Klebsiella* phage ZCKP8. Additionally, the proteomic tree grouped the phage ZCKP2 with unclassified siphoviruses in a separate clade from siphoviruses of the family *Drexlerviridae* (Fig. 8). The genomes of the closely related phages (ViPTree score *S*_*G*_ > 0.64) were aligned and compared to ZCKP2 (Fig. 9**).** The whole genome comparison highlighted the differences between the genomes, and particularly, between phage ZCKP2 and the closest phage (ZCKP8). Therefore, phage ZCKP2 may be novel based on results from whole-genome comparison and VIRIDIC’s intergenomic similarity.

The pan-genome analysis of phage ZCKP2 was conducted against closely related phages, representatives from the family *Drexlerviridae* and outgroup phages (myoviruses and podoviruses) (Supplementary Tables S2-S6). The pan genome analysis revealed that phage ZCKP2 and closely related phages of *S*_*G*_ above 0.5 shared 24 orthologous genes. As summarized in Supplementary Table S7, the number of orthologs increased with higher *S*_*G*_, reaching a maximum of 69 in the case of the closest phage, ZCKP8 (*S*_*G*_ = 0.92). Conversely, the number of orthologs dropped to seven when phage ZCKP2 was compared to phages from the family *Drexlerviridae*.

**Fig. 10:**
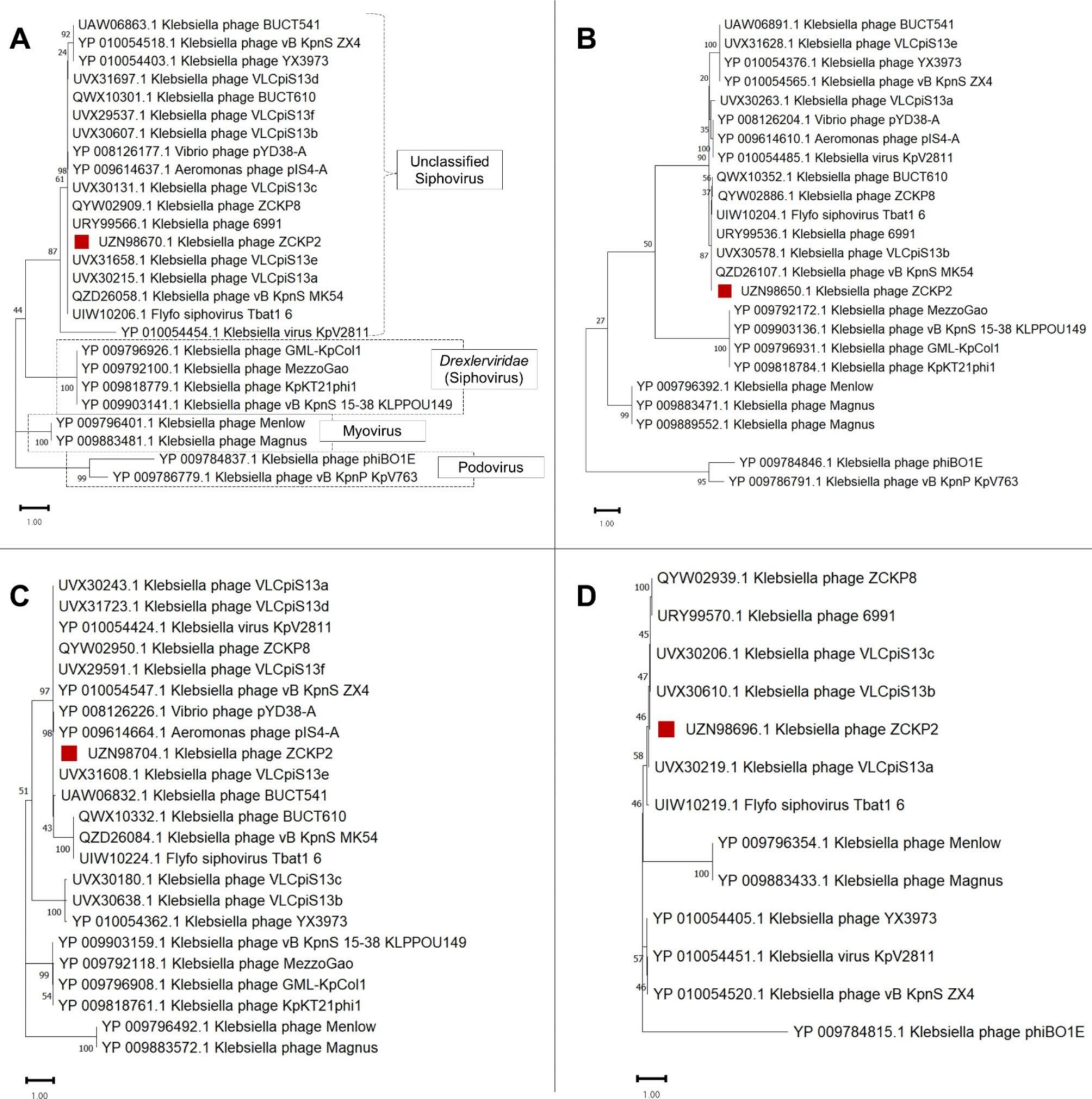
Phylogenetic tree of the aligned amino acid sequence of signature proteins, **(A)** major capsid protein, **(B)** terminase large subunit, **(C)** single-strand DNA-binding protein, and **(D)** DNA polymerase III. The analysis was conducted by the Maximum likelihood method with a bootstrap of 100 replicates, in MEGA11.

Based on the pan-genome analysis, the proteins of the most prominent signature genes were selected to conduct further phylogenetic analysis (Fig. 10A-D). The signature genes included major capsid protein, terminase large subunit, single-strand DNA-binding protein, and DNA polymerase III. In the four trees, phage ZCKP2 was clustered with other unclassified siphoviruses, but particularly *Klebsiella* phage ZCKP8, phage 6991, phage VLCpiS13a, phage VLCpiS13b, and phage VLCpiS13c were common to all clusters. Representatives of the family *Drexlerviridae* were separately clustered from ZCKP2 in all inferred protein-based phylogenetic trees. Consequently, phage ZCKP2 most likely represents a new family with other unclassified siphoviruses and shares the same genus as ZCKP8.

## Discussion

*Klebsiella pneumoniae* is an important opportunistic pathogen that regularly causes nosocomial infections and contributes to substantial morbidity and mortality. Currently, *K. pneumoniae* is showing a high resistance to a wide-ranging spectrum of drugs including beta-lactam antibiotics, fluoroquinolones, and aminoglycosides [41, 73, 94]. Commonly, antimicrobial resistance is associated with the proliferation of transmissible plasmids and the acquisition of resistance genes that generally occur via horizontal gene transfer, which may also include virulence factors [74, 94].

Bacteriophage therapy is one such approach that can be used as an alternative to antibiotics. Conventionally, phage therapy relies on the use of naturally occurring bacterial parasites which are incapable of reproducing on their own (i.e., they are non-living) and are entirely dependent on a bacterial host for their existence by infecting and lysing them [75]. Their therapeutic potential in medicine to control MDR pathogens is due to their specificity and potency in inducing lethal effects in the host bacterium by cell lysis [76]. Interestingly, phage vB_Kpn_ZC2 (ZCKP2) infected 7 out of 30 bacterial strains, demonstrating a relatively narrow host range. However, ZCKP2 has shown that the phage may lyse several *Klebsiella* strains. The most vulnerable bacterial host is intended to be used in the classic isolation method for enrichment. However, current research indicates that using a large number of hosts during the isolation process increases the likelihood of separating phages with greater host ranges.

The evaluation of the phage growth curve is considered the essential part to be demonstrated when it is applied as a therapeutic agent. The short latent period and large burst size (> 200 PFU/cell) of phage ZCKP2 are compared to a few published *Klebsiella* siphoviruses [22, 77] and also, a few phages had medium burst size [39, 78, 79].

Time-killing curves were done to study the antibacterial activity of ZCKP2 phage against its host KP/08 by infecting the bacteria at exponential phase for 325 min with phage at different MOIs in a shaking condition to achieve a faster rate of bacterial growth and the homogeneous distribution of the nutrition [80]. The curves showed that the ZCKP2 phage inhibited the bacterial growth in a MOI-dependent manner, where the higher MOIs showed the highest reduction value and the bacteria started to display an increase in the O.D reading, which might reflect the probable phage resistance. These results are consistent with the previous phages, in which the ZCKP2 phage displayed similar activity as the ZCKP1 [52], ZCKP8 [41], vB_KpnS_Kp13 [81] and P545 [82], but different activity from vB_KpnS_MK54 that employed a different methodology for determining the optimal MOI. Unlike, ZCKP1, vB_KpnS_Kp13, P545 and ZCKP2 phages, vB_KpnS_MK54 phage has an optimal MOI of 0.01. Respectively, further investigations are needed to study and standardize the methodology of determining the optimal MOI [83].

The resistance to environmental stress assay was performed to investigate the phage application of primary conditions and prospects [84]. Phage stability following exposure to varying temperatures and pH was determined. The phage was stable at -20 °C and 4 °C. Although the titer of the phages was slightly reduced after 4 h of exposure at 40 °C, this phage was stable at temperatures ranging from 20 °C to 50 °C. Incubation at more than 60 °C for 4 h was lethal to the phage, thus completely inactivating them. Such characteristics aligned with the results reported in a previous study with the phage VpKK5. The phage BPA43 was tested for its pH stability and the phage was also found to be stable from pH 4.0 to 9.0 and was most active at pH 5.0 whilst it lost its activity completely at pH 3.0, 11, 12, and 13 [85].

The whole genome analysis facilitates the characterization of novel phages and readily fills the gaps resulting from in vitro analysis. The genomic characterization identified a cluster of two adjacent genes (ORFs 14 and 15) encoding putative holin and lysozyme. Holins form holes in the inner membrane of the bacterial cytoplasmic membrane; these holes allow lysozymes to leak into the periplasmic space; consequently, the lysozymes can reach and effectively break down the bacterial rigid barrier ‘peptidoglycan’ [86]. The predicted cluster of the genes encoding holin and lysozyme reveals the possible mechanism by which ZCKP2 lyses the infected bacterial cells to free the newly formed progeny at the end of the lytic cycle. In silico analysis of the phage did not detect any genes related to lysogeny, antibiotic resistance or bacterial virulence. Accordingly, ZCKP2 genomic analysis strongly suggests the safety and applicability of using a phage as a therapeutic agent [37].

Identifying regions of transmembrane in the putative proteins would indicate possible functions of these proteins. DeepTMHMM tool predicted two hydrophobic transmembrane domains in the putative holin (ORF 15, 81 amino acids) that comply with the topology of class II holins. Class II holins are characterized by double transmembrane and 60 to 185 amino acid residues [87]. Likewise, the putative tail length measuring protein (ORF 35) had two transmembrane domains. The latter prediction complies with this protein function as it facilitates the transfer of viral DNA to the infected bacterial cytoplasm through forming a channel within the bacterial cell membranes [88]. Also, double transmembrane topology was predicted in the hypothetical protein (ORF 28), suggesting that this protein could be a novel antimicrobial [89]. Future research on ZCKP2 would isolate and investigate this hypothetical protein activity.

Different approaches were applied for the phylogenetic analysis of ZCKP2, and all these approaches had quite similar results. VIRIDIC classified ZCKP2 is the same genus as ZCKP8, but the intergenomic similarity was below the species clustering threshold. VIRIDIC classification is highly reliable since it follows the algorithm used by ICTV [68]. Similarly, proteomic tree phylogeny inferred that ZCKP2 is closely related to ZCKP8 and other siphoviruses, but distantly related to members of family *Drexlerviridae*. Proteomic trees reveal information about the evolutionary history of phages [90, 91], which is useful for studying more distant relationships [92, 95]. According to the International Committee on Taxonomy of Viruses (ICTV) 2018–2019 update, the host type and genomic characteristics (GC content, genome size, number of coding sequences) of the family *Drexlerviridae* [93] are similar to those of phage ZCKP2; however, proteomic and pan-genome evolutionary analyses clustered the phage in a different unrated family.

## Conclusion

Phage therapy gains more scientific attention as a potential alternative to antibiotics. Here, the paper evaluated the potential activity and safety of phage vB_Kpn_ZC2 (ZCKP2) upon employing it to control the growth of *K. pneumoniae*. The findings suggest that phage ZCKP2 is a potential candidate for further study in vivo to confirm its safety and efficiency in the field of phage therapy. In addition, further studies can be done to augment the phage *K. pneumoniae* infections with other phages (a phage cocktail) or antibiotics to increase its host range and target more bacteria in different applications.

## Electronic supplementary material

Below is the link to the electronic supplementary material.

Supplementary Material 1

## Data Availability

The dataset presented in this study can be found in NCBI GenBank. The 16s rRNA sequence was deposited under the accession number OP410967.1. In addition, the annotated genome sequence of the phage ZCKP2 was deposited under the accession numbers GenBank Acc. No. NC_071151.
