# Correction: Morphological, biological, and genomic characterization of *Klebsiella pneumoniae* phage vB_Kpn_ZC2

**DOI:** 10.1186/s12985-023-02083-2

**Published:** 2023-06-05

**Authors:** Mohamed S. Fayez, Toka A. Hakim, Bishoy Maher Zaki, Salsabil Makky, Mohamed Abdelmoteleb, Kareem Essam, Anan Safwat, Abdallah S. Abdelsattar, Ayman El-Shibiny

**Affiliations:** 1 Center for Microbiology and Phage Therapy, Zewail City of Science and Technology, Giza, 12578 Egypt; 2 Microbiology and Immunology Department, Faculty of Pharmacy, October University for Modern Sciences and Arts (MSA), Giza, 11787 Egypt; 3 Department of Botany, Faculty of Science, Mansoura University, Mansoura, 35516 Egypt; 4 Faculty of Environmental Agricultural Sciences, Arish University, Arish, 45511 Egypt

**Correction: Virology Journal (2023) 20:86** **https://doi.org/10.1186/s12985-023-02034-x**

Following publication of the original article [1], the authors informed us that the grant number has been missing from the Funding section. The correct Funding is given below:

**Funding**

Open access funding provided by The Science, Technology & Innovation Funding Authority (STDF) grant #41909 in cooperation with The Egyptian Knowledge Bank (EKB).

The original article has been corrected.
