# Supplementary material for: Morphological, biological, and genomic characterization of Klebsiella pneumoniae phage vB_Kpn_ZC2
Source: Virol J. 2023 May 3;20:86. doi: 10.1186/s12985-023-02034-x (PMC10158348; doi:10.1186/s12985-023-02034-x)
Supplement: Supplementary file 1 — Supplementary Material 1 [file 12985_2023_2034_MOESM1_ESM.pdf]

Table S1. The list of the putative protein-coding genes

ORF#	Strand	Frame	CDS Position	Translation	Best annotated protein
ORF1	+	1	283..468	186 | 61	Hypothetical protein
ORF2	+	1	484..762	279 | 92	Hypothetical protein
ORF3	+	1	769..1152	384 | 127	Hypothetical protein
ORF4	+	2	1286..1789	504 | 167	EaA-like protein
ORF5	+	2	1865..2713	849 | 282	Hypothetical protein
ORF6	+	3	2964..3158	195 | 64	Hypothetical protein
ORF7	+	3	3261..3680	420 | 139	Hypothetical protein
ORF8	+	2	3800..5242	1443 | 480	Large terminase subunit
ORF9	+	3	6261..6500	240 | 79	Hypothetical protein
ORF10	+	3	6846..7067	222 | 73	Hypothetical protein
ORF11	+	2	7274..7519	246 | 81	Hypothetical protein
ORF12	+	1	7567..8961	1395 | 464	Portal (connector) protein
ORF13	+	1	9733..10680	948 | 315	Head decoration protein
ORF14	+	3	12195..12632	438 | 145	Lysozyme
ORF15	+	2	12629..12874	246 | 81	Holin protein
ORF16	+	3	12849..13343	495 | 164	Tail protein
ORF17	+	2	14852..15067	216 | 71	Hypothetical protein
ORF18	+	1	15151..15342	192 | 63	Hypothetical protein
ORF19	+	2	15560..15796	237 | 78	Zinc-binding domain of primase-helicase
ORF20	+	2	16244..16534	291 | 96	Hypothetical protein
ORF21	+	2	16535..16879	345 | 114	Hypothetical protein
ORF22	+	1	16894..17046	153 | 50	Hypothetical protein
ORF23	+	1	17062..18462	1401 | 466	Hypothetical protein
ORF24	+	3	18462..18926	465 | 154	Capsid decoration protein
ORF25	+	2	18938..20026	1089 | 362	Major coat protein
ORF26	+	2	20069..20263	195 | 64	Hypothetical protein
ORF27	+	1	20293..21123	831 | 276	Hypothetical protein
ORF28	+	1	23863..24150	288 | 95	Hypothetical protein
ORF29	+	2	24059..24520	462 | 153	Hypothetical protein
ORF30	+	3	24639..24983	345 | 114	Hypothetical protein
ORF31	+	3	24987..25403	417 | 138	Hypothetical protein
ORF32	+	2	25400..25783	384 | 127	Hypothetical protein
ORF33	+	1	26317..27069	753 | 250	Major tail subunit
ORF34	+	1	28729..29436	708 | 235	Hypothetical protein
ORF35	+	3	29436..32717	3282 | 1093	Tail length tape measure protein
ORF36	+	2	32717..33187	471 | 156	Minor tail protein
ORF37	+	2	33599..34078	480 | 159	Tail assembly protein
ORF38	+	3	34026..36506	2481 | 826	Putative tail protein
ORF39	+	2	36536..39028	2493 | 830	Putative tail fiber protein
ORF40	+	2	45053..46006	954 | 317	DNA primase
ORF41	+	1	46003..46320	318 | 105	Hypothetical protein
ORF42	-	2	5822..5256	567 | 188	Hypothetical protein
ORF43	-	3	6172..5882	291 | 96	Hypothetical protein

ORF44	-	2	11678..10815	864 | 287	Hypothetical protein
ORF45	-	1	12096..11806	291 | 96	Hypothetical protein
ORF46	-	1	13617..13402	216 | 71	Hypothetical protein
ORF47	-	3	13972..13748	225 | 74	Hypothetical protein
ORF48	-	1	14361..13972	390 | 129	Hypothetical protein
ORF49	-	3	14755..14555	201 | 66	Holin
ORF50	-	2	21335..21153	183 | 60	Hypothetical protein
ORF51	-	3	21916..21338	579 | 192	DNA polymerase III beta subunit
ORF52	-	1	22047..21874	174 | 57	Hypothetical protein
ORF53	-	2	22391..22044	348 | 115	Hypothetical protein
ORF54	-	3	23242..22391	852 | 283	DNA binding protein
ORF55	-	3	23650..23327	324 | 107	Hypothetical protein
ORF56	-	1	27777..27109	669 | 222	Gp42
ORF57	-	2	28028..27777	252 | 83	Hypothetical protein
ORF58	-	3	28510..28025	486 | 161	HNH endonuclease
ORF59	-	3	39805..39302	504 | 167	Single-stranded DNA-binding protein
ORF60	-	3	40474..39815	660 | 219	Recombinase
ORF61	-	1	41454..40498	957 | 318	Exonuclease
ORF62	-	2	41900..41457	444 | 147	Hypothetical protein
ORF63	-	3	42364..41948	417 | 138	Hypothetical protein
ORF64	-	2	44258..42351	1908 | 635	DNA helicase
ORF65	-	2	45005..44853	153 | 50	Hypothetical protein
ORF66	-	3	45040..44267	774 | 257	HNH endonuclease
ORF67	-	1	46296..46021	276 | 91	ASCH domain-containing protein
ORF68	-	2	47852..46296	1557 | 518	Hypothetical protein
ORF69	-	3	48085..47849	237 | 78	Hypothetical protein

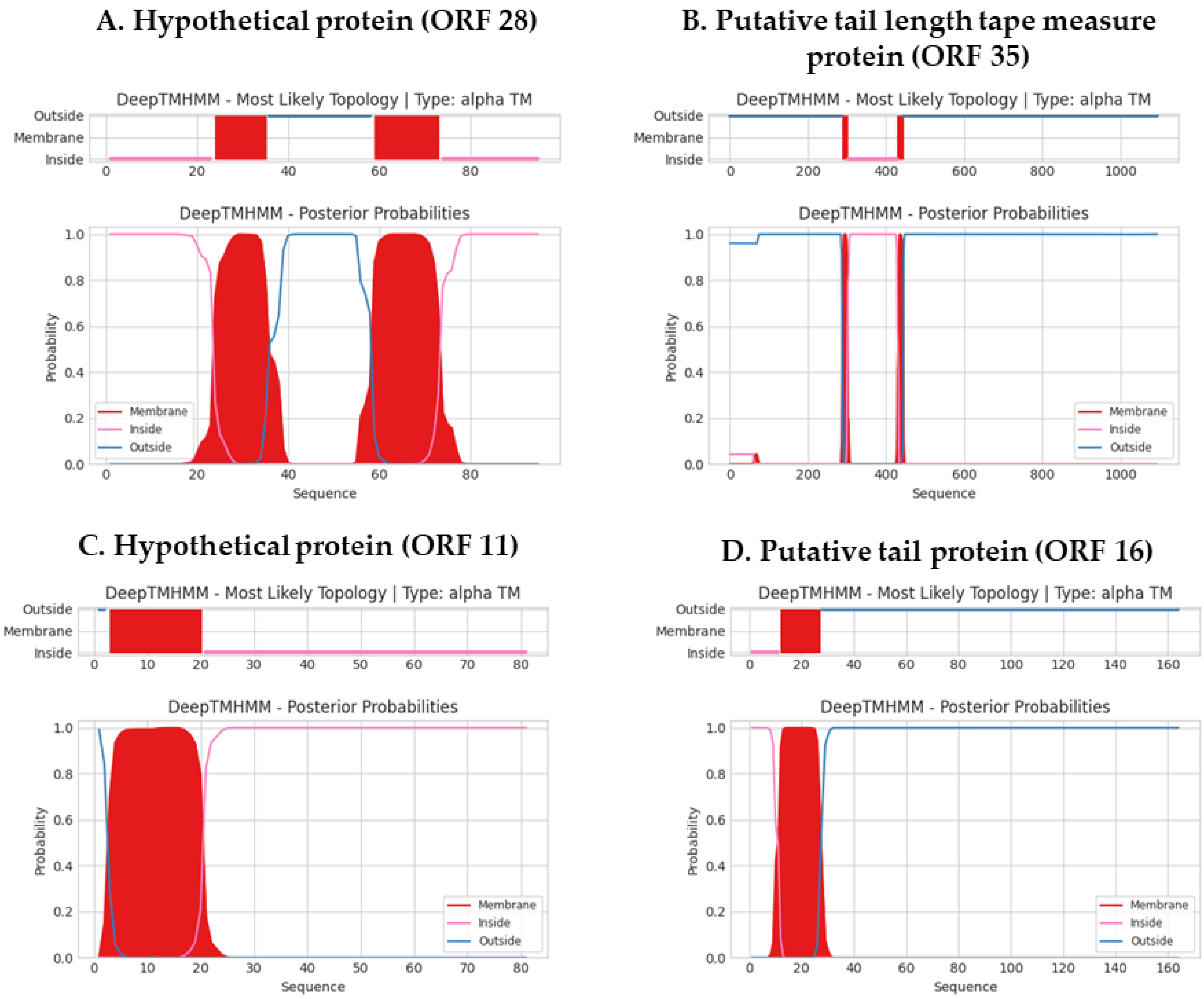

Figure S1. Predicted transmembrane topology using the DeepTMHMM tool of four putative proteins (A: ORF 28, B: ORF 35, C: ORF 11, D: ORF 16). Red blocks represent the predicted transmembrane domains, while the pink line and blue line represent the domains inside and outside the membrane, respectively. The Y-axis represents the prediction probability, while the X-axis represents the amino acids sequence position

Table S2. Pan-genome analysis of ZCKP2 and closely related phages (S_G ≥0.5)

The number of homologs in each column is: 24

Klebsiella phage ZCKP2 OP481875	Klebsiella phage ZCKP8 MZ440881	Vibrio phage pYD38-A NC_021534	Aeromonas phage pIS4-A NC_042037	Flyfo siphovirus Tbat1_6 OL617041	Klebsiella phage vB_KpnS_MK54 MW119258	Klebsiella phage BUCT610 MZ318367	Klebsiella phage BUCT541 MZ836210	Klebsiella phage vB_KpnS_ZX4 NC_054654	Klebsiella phage YX3973 NC_054652	Klebsiella virus KpV2811 NC_054653	Klebsiella phage 6991 OL362277	Klebsiella phage VLCpiS13f ON602730	Klebsiella phage VLCpiS13c ON602737	Klebsiella phage VLCpiS13a ON602738	Klebsiella phage VLCpiS13b ON602744	Klebsiella phage VLCpiS13e ON602759	Klebsiella phage VLCpiS13d ON602760
PI:UZN98670.1 Product:major coat protein	PI:QYW02909.1 Product:major coat protein	PI:YP_008126177.1 Product:virion structural protein	PI:YP_009614637.1 Product:virion structural protein	PI:UIW10206.1 Product:protein of unknown function DUF6260	PI:QZD26058.1 Product:major capsid protein	PI:QWX10301.1 Product:protein of unknown function DUF2184	PI:UAW06863.1 Product:coat protein	PI:YP_010054518.1 Product:virion structural protein	PI:YP_010054403.1 Product:virion structural protein	PI:YP_010054454.1 Product:virion structural protein	PI:URY99566.1 Product:coat protein	PI:UVX29537.1 Product:virion structural protein	PI:UVX30131.1 Product:virion structural protein	PI:UVX30215.1 Product:virion structural protein	PI:UVX30607.1 Product:virion structural protein	PI:UVX31658.1 Product:virion structural protein	PI:UVX31697.1 Product:virion structural protein
PI:UZN98643.1 Product:hypothetical protein	PI:QYW02877.1 Product:hypothetical protein	PI:YP_008126213.1 Product:hypothetical protein	PI:YP_009614601.1 Product:hypothetical protein	PI:UIW10248.1 Product:hypothetical protein	PI:QZD26098.1 Product:hypothetical protein	PI:QWX10344.1 Product:hypothetical protein	PI:UAW06819.1 Product:hypothetical protein	PI:YP_010054559.1 Product:hypothetical protein	PI:YP_010054370.1 Product:hypothetical protein	PI:YP_010054492.1 Product:hypothetical protein	PI:URY99606.1 Product:hypothetical protein	PI:UVX29578.1 Product:hypothetical protein	PI:UVX30173.1 Product:hypothetical protein	PI:UVX30255.1 Product:hypothetical protein	PI:UVX30648.1 Product:hypothetical protein	PI:UVX31619.1 Product:hypothetical protein	PI:UVX31735.1 Product:hypothetical protein
PI:UZN98688.1 Product:hypothetical protein	PI:QYW02928.1 Product:hypothetical protein	PI:YP_008126203.1 Product:hypothetical protein	PI:YP_009614611.1 Product:hypothetical protein	PI:UIW10242.1 Product:hypothetical protein	PI:QZD26108.1 Product:hypothetical protein	PI:QWX10271.1 Product:hypothetical protein	PI:UAW06890.1 Product:hypothetical protein	PI:YP_010054494.1 Product:hypothetical protein	PI:YP_010054377.1 Product:hypothetical protein	PI:YP_010054484.1 Product:hypothetical protein	PI:URY99538.1 Product:hypothetical protein	PI:UVX29568.1 Product:hypothetical protein	PI:UVX30161.1 Product:hypothetical protein	PI:UVX30264.1 Product:hypothetical protein	PI:UVX30580.1 Product:hypothetical protein	PI:UVX31630.1 Product:hypothetical protein	PI:UVX31665.1 Product:hypothetical protein
PI:UZN98651.1 Product:hypothetical protein	PI:QYW02887.1 Product:hypothetical protein	PI:YP_008126202.1 Product:hypothetical protein	PI:YP_009614612.1 Product:hypothetical protein	PI:UIW10258.1 Product:hypothetical protein	PI:QZD26109.1 Product:hypothetical protein	PI:QWX10272.1 Product:hypothetical protein	PI:UAW06889.1 Product:hypothetical protein	PI:YP_010054495.1 Product:hypothetical protein	PI:YP_010054378.1 Product:hypothetical protein	PI:YP_010054483.1 Product:hypothetical protein	PI:URY99539.1 Product:hypothetical protein	PI:UVX29567.1 Product:hypothetical protein	PI:UVX30160.1 Product:hypothetical protein	PI:UVX30265.1 Product:hypothetical protein	PI:UVX30581.1 Product:hypothetical protein	PI:UVX31631.1 Product:hypothetical protein	PI:UVX31666.1 Product:hypothetical protein
PI:UZN98654.1 Product:portal (connector) protein	PI:QYW02893.1 Product:portal (connector) protein	PI:YP_008126197.1 Product:portal protein	PI:YP_009614617.1 Product:portal protein	PI:UIW10203.1 Product:protein of unknown function DUF1073	PI:QZD26115.1 Product:portal protein	PI:QWX10279.1 Product:portal protein	PI:UAW06885.1 Product:hypothetical protein	PI:YP_010054497.1 Product:portal protein	PI:YP_010054381.1 Product:portal protein	PI:YP_010054478.1 Product:portal protein	PI:URY99544.1 Product:portal (connector) protein	PI:UVX29561.1 Product:portal protein	PI:UVX30155.1 Product:portal protein	PI:UVX30272.1 Product:portal protein	PI:UVX30585.1 Product:portal protein	PI:UVX31635.1 Product:portal protein	PI:UVX31673.1 Product:portal protein
PI:UZN98656.1 Product:head decoration protein	PI:QYW02895.1 Product:head decoration protein	PI:YP_008126196.1 Product:head morphogenesis	PI:YP_009614618.1 Product:head morphogenesis	PI:UIW10207.1 Product:hypothetical protein	PI:QZD26116.1 Product:minor capsid protein	PI:QWX10280.1 Product:hypothetical protein	PI:UAW06884.1 Product:hypothetical protein	PI:YP_010054498.1 Product:head morphogenesis	PI:YP_010054384.1 Product:head morphogenesis	PI:YP_010054477.1 Product:head morphogenesis	PI:URY99545.1 Product:hypothetical protein	PI:UVX29560.1 Product:head morphogenesis	PI:UVX30153.1 Product:head morphogenesis	PI:UVX30273.1 Product:head morphogenesis	PI:UVX30586.1 Product:head morphogenesis	PI:UVX31636.1 Product:head morphogenesis	PI:UVX31674.1 Product:head morphogenesis
PI:UZN98690.1 Product:hypothetical protein	PI:QYW02932.1 Product:hypothetical protein	PI:YP_008126193.1 Product:hypothetical protein	PI:YP_009614621.1 Product:hypothetical protein	PI:UIW10244.1 Product:hypothetical protein	PI:QZD26120.1 Product:hypothetical protein	PI:QWX10284.1 Product:hypothetical protein	PI:UAW06881.1 Product:hypothetical protein	PI:YP_010054502.1 Product:hypothetical protein	PI:YP_010054387.1 Product:hypothetical protein	PI:YP_010054472.1 Product:hypothetical protein	PI:URY99549.1 Product:hypothetical protein	PI:UVX29557.1 Product:hypothetical protein	PI:UVX30150.1 Product:hypothetical protein	PI:UVX30276.1 Product:hypothetical protein	PI:UVX30589.1 Product:hypothetical protein	PI:UVX31640.1 Product:hypothetical protein	PI:UVX31677.1 Product:hypothetical protein
PI:UZN98657.1 Product:lysozyme	PI:QYW02896.1 Product:lysozyme	PI:YP_008126192.1 Product:endolysin	PI:YP_009614622.1 Product:endolysin	PI:UIW10247.1 Product:hypothetical protein	PI:QZD26122.1 Product:lysozyme	PI:QWX10285.1 Product:lysozyme	PI:UAW06880.1 Product:lysozyme	PI:YP_010054503.1 Product:endolysin	PI:YP_010054388.1 Product:endolysin	PI:YP_010054471.1 Product:endolysin	PI:URY99550.1 Product:lysozyme R	PI:UVX29556.1 Product:endolysin	PI:UVX30149.1 Product:endolysin	PI:UVX30277.1 Product:endolysin	PI:UVX30591.1 Product:endolysin	PI:UVX31641.1 Product:endolysin	PI:UVX31678.1 Product:endolysin
PI:UZN98693.1 Product:hypothetical protein	PI:QYW02936.1 Product:hypothetical protein	PI:YP_008126189.1 Product:hypothetical protein	PI:YP_009614625.1 Product:hypothetical protein	PI:UIW10220.1 Product:hypothetical protein	PI:QZD26047.1 Product:hypothetical protein	PI:QWX10290.1 Product:hypothetical protein	PI:UAW06874.1 Product:hypothetical protein	PI:YP_010054507.1 Product:hypothetical protein	PI:YP_010054392.1 Product:hypothetical protein	PI:YP_010054466.1 Product:hypothetical protein	PI:URY99554.1 Product:hypothetical protein	PI:UVX29549.1 Product:hypothetical protein	PI:UVX30143.1 Product:hypothetical protein	PI:UVX30283.1 Product:hypothetical protein	PI:UVX30595.1 Product:hypothetical protein	PI:UVX31647.1 Product:hypothetical protein	PI:UVX31683.1 Product:hypothetical protein
PI:UZN98694.1 Product:holin	PI:QYW02937.1 Product:holin	PI:YP_008126187.1 Product:hypothetical protein	PI:YP_009614627.1 Product:hypothetical protein	PI:UIW10267.1 Product:hypothetical protein	PI:QZD26049.1 Product:hypothetical protein	PI:QWX10291.1 Product:hypothetical protein	PI:UAW06873.1 Product:hypothetical protein	PI:YP_010054508.1 Product:hypothetical protein	PI:YP_010054393.1 Product:holin	PI:YP_010054465.1 Product:hypothetical protein	PI:URY99556.1 Product:hypothetical protein	PI:UVX29547.1 Product:hypothetical protein	PI:UVX30141.1 Product:hypothetical protein	PI:UVX30285.1 Product:hypothetical protein	PI:UVX30596.1 Product:hypothetical protein	PI:UVX31649.1 Product:hypothetical protein	PI:UVX31684.1 Product:hypothetical protein
PI:UZN98660.1 Product:hypothetical protein	PI:QYW02899.1 Product:hypothetical protein	PI:YP_008126186.1 Product:hypothetical protein	PI:YP_009614628.1 Product:hypothetical protein	PI:UIW10263.1 Product:hypothetical protein	PI:QZD26050.1 Product:hypothetical protein	PI:QWX10292.1 Product:hypothetical protein	PI:UAW06872.1 Product:hypothetical protein	PI:YP_010054509.1 Product:hypothetical protein	PI:YP_010054394.1 Product:hypothetical protein	PI:YP_010054463.1 Product:hypothetical protein	PI:URY99557.1 Product:hypothetical protein	PI:UVX29546.1 Product:hypothetical protein	PI:UVX30140.1 Product:hypothetical protein	PI:UVX30286.1 Product:hypothetical protein	PI:UVX30597.1 Product:hypothetical protein	PI:UVX31650.1 Product:hypothetical protein	PI:UVX31685.1 Product:hypothetical protein
PI:UZN98664.1 Product:hypothetical protein	PI:QYW02903.1 Product:hypothetical protein	PI:YP_008126182.1 Product:MarR family transcription regulator	PI:YP_009614632.1 Product:MarR family transcription regulator	PI:UIW10226.1 Product:hypothetical protein	PI:QZD26053.1 Product:hypothetical protein	PI:QWX10296.1 Product:hypothetical protein	PI:UAW06868.1 Product:transcription factor	PI:YP_010054513.1 Product:MarR family transcription regulator	PI:YP_010054398.1 Product:MarR family transcription regulator	PI:YP_010054459.1 Product:MarR family transcription regulator	PI:URY99561.1 Product:hypothetical protein	PI:UVX29542.1 Product:MarR family transcription regulator	PI:UVX30136.1 Product:MarR family transcription regulator	PI:UVX30210.1 Product:MarR family transcription regulator	PI:UVX30601.1 Product:MarR family transcription regulator	PI:UVX31653.1 Product:MarR family transcription regulator	PI:UVX31689.1 Product:MarR family transcription regulator
PI:UZN98666.1 Product:hypothetical protein	PI:QYW02905.1 Product:hypothetical protein	PI:YP_008126180.1 Product:hypothetical protein	PI:YP_009614634.1 Product:hypothetical protein	PI:UIW10237.1 Product:hypothetical protein	PI:QZD26055.1 Product:hypothetical protein	PI:QWX10298.1 Product:hypothetical protein	PI:UAW06866.1 Product:hypothetical protein	PI:YP_010054515.1 Product:hypothetical protein	PI:YP_010054400.1 Product:hypothetical protein	PI:YP_010054457.1 Product:hypothetical protein	PI:URY99563.1 Product:hypothetical protein	PI:UVX29540.1 Product:hypothetical protein	PI:UVX30134.1 Product:hypothetical protein	PI:UVX30212.1 Product:hypothetical protein	PI:UVX30604.1 Product:hypothetical protein	PI:UVX31655.1 Product:hypothetical protein	PI:UVX31694.1 Product:hypothetical protein
PI:UZN98668.1 Product:hypothetical protein	PI:QYW02907.1 Product:hypothetical protein	PI:YP_008126179.1 Product:hypothetical protein	PI:YP_009614635.1 Product:hypothetical protein	PI:UIW10205.1 Product:hypothetical protein	PI:QZD26056.1 Product:prohead core protein serine protease	PI:QWX10299.1 Product:coat protein	PI:UAW06865.1 Product:hypothetical protein	PI:YP_010054516.1 Product:coat protein	PI:YP_010054401.1 Product:hypothetical protein	PI:YP_010054456.1 Product:hypothetical protein	PI:URY99564.1 Product:coat protein	PI:UVX29539.1 Product:hypothetical protein	PI:UVX30133.1 Product:hypothetical protein	PI:UVX30213.1 Product:hypothetical protein	PI:UVX30605.1 Product:hypothetical protein	PI:UVX31656.1 Product:hypothetical protein	PI:UVX31695.1 Product:hypothetical protein
PI:UZN98699.1 Product:DNA binding protein	PI:QYW02942.1 Product:putative transcriptional regulator	PI:YP_008126170.1 Product:ParB-like partition protein	PI:YP_009614644.1 Product:ParB-like partition protein	PI:UIW10212.1 Product:KorB domain protein	PI:QZD26064.1 Product:putative transcriptional regulator	PI:QWX10308.1 Product:hypothetical protein	PI:UAW06856.1 Product:hypothetical protein	PI:YP_010054524.1 Product:ParB-like partition protein	PI:YP_010054409.1 Product:ParB-like partition protein	PI:YP_010054447.1 Product:ParB-like partition protein	PI:URY99573.1 Product:hypothetical protein	PI:UVX29532.1 Product:ParB-like partition protein	PI:UVX30203.1 Product:ParB-like partition protein	PI:UVX30222.1 Product:hypothetical protein	PI:UVX30613.1 Product:ParB-like partition protein	PI:UVX31664.1 Product:ParB-like partition protein	PI:UVX31702.1 Product:hypothetical protein
PI:UZN98674.1 Product:hypothetical protein	PI:QYW02913.1 Product:hypothetical protein	PI:YP_008126167.1 Product:hypothetical protein	PI:YP_009614647.1 Product:hypothetical protein	PI:UIW10235.1 Product:hypothetical protein	PI:QZD26069.1 Product:head to tail adaptor	PI:QWX10312.1 Product:hypothetical protein	PI:UAW06852.1 Product:hypothetical protein	PI:YP_010054528.1 Product:hypothetical protein	PI:YP_010054412.1 Product:hypothetical protein	PI:YP_010054443.1 Product:hypothetical protein	PI:URY99577.1 Product:hypothetical protein	PI:UVX29527.1 Product:hypothetical protein	PI:UVX30199.1 Product:hypothetical protein	PI:UVX30224.1 Product:hypothetical protein	PI:UVX30617.1 Product:hypothetical protein	PI:UVX31589.1 Product:hypothetical protein	PI:UVX31704.1 Product:hypothetical protein
PI:UZN98675.1 Product:hypothetical protein	PI:QYW02915.1 Product:hypothetical protein	PI:YP_008126166.1 Product:hypothetical protein	PI:YP_009614648.1 Product:hypothetical protein	PI:UIW10236.1 Product:hypothetical protein	PI:QZD26070.1 Product:minor capsid protein	PI:QWX10313.1 Product:hypothetical protein	PI:UAW06850.1 Product:minor capsid protein	PI:YP_010054530.1 Product:hypothetical protein	PI:YP_010054413.1 Product:hypothetical protein	PI:YP_010054441.1 Product:hypothetical protein	PI:URY99579.1 Product:hypothetical protein	PI:UVX29526.1 Product:hypothetical protein	PI:UVX30197.1 Product:hypothetical protein	PI:UVX30226.1 Product:hypothetical protein	PI:UVX30618.1 Product:hypothetical protein	PI:UVX31591.1 Product:hypothetical protein	PI:UVX31705.1 Product:hypothetical protein
PI:UZN98677.1 Product:hypothetical protein	PI:QYW02917.1 Product:hypothetical protein	PI:YP_008126240.1 Product:hypothetical protein	PI:YP_009614650.1 Product:hypothetical protein	PI:UIW10234.1 Product:hypothetical protein	PI:QZD26072.1 Product:tail completion protein	PI:QWX10315.1 Product:hypothetical protein	PI:UAW06848.1 Product:hypothetical protein	PI:YP_010054532.1 Product:hypothetical protein	PI:YP_010054415.1 Product:hypothetical protein	PI:YP_010054439.1 Product:hypothetical protein	PI:URY99581.1 Product:hypothetical protein	PI:UVX29524.1 Product:hypothetical protein	PI:UVX30195.1 Product:hypothetical protein	PI:UVX30228.1 Product:hypothetical protein	PI:UVX30620.1 Product:hypothetical protein	PI:UVX31593.1 Product:hypothetical protein	PI:UVX31707.1 Product:hypothetical protein
PI:UZN98678.1 Product:major tail subunit	PI:QYW02919.1 Product:major tail subunit	PI:YP_008126238.1 Product:major tail protein	PI:YP_009614652.1 Product:major tail protein	PI:UIW10213.1 Product:Immunoglobulin domain protein	PI:QZD26073.1 Product:major tail subunit	PI:QWX10318.1 Product:hypothetical protein	PI:UAW06845.1 Product:major tail protein	PI:YP_010054534.1 Product:major tail protein	PI:YP_010054352.1 Product:major tail protein	PI:YP_010054437.1 Product:major tail protein	PI:URY99583.1 Product:hypothetical protein	PI:UVX29523.1 Product:major tail protein	PI:UVX30194.1 Product:major tail protein	PI:UVX30229.1 Product:major tail protein	PI:UVX30621.1 Product:major tail protein	PI:UVX31595.1 Product:major tail protein	PI:UVX31708.1 Product:major tail protein
PI:UZN98679.1 Product:hypothetical protein	PI:QYW02920.1 Product:hypothetical protein	PI:YP_008126234.1 Product:DUF6246 family protein	PI:YP_009614656.1 Product:DUF6246 family protein	PI:UIW10216.1 Product:protein of unknown function DUF6246	PI:QZD26077.1 Product:hypothetical protein	PI:QWX10324.1 Product:hypothetical protein	PI:UAW06839.1 Product:hypothetical protein	PI:YP_010054540.1 Product:DUF6246 family protein	PI:YP_010054354.1 Product:DUF6246 family protein	PI:YP_010054430.1 Product:DUF6246 family protein	PI:URY99588.1 Product:hypothetical protein	PI:UVX29518.1 Product:hypothetical protein	PI:UVX30189.1 Product:hypothetical protein	PI:UVX30234.1 Product:hypothetical protein	PI:UVX30626.1 Product:hypothetical protein	PI:UVX31600.1 Product:hypothetical protein	PI:UVX31714.1 Product:hypothetical protein
PI:UZN98680.1 Product:tail length tape measure protein	PI:QYW02921.1 Product:tail length tape measure protein	PI:YP_008126233.1 Product:tail length tape measure protein	PI:YP_009614657.1 Product:tail length tape measure protein	PI:UIW10199.1 Product:tape measure protein	PI:QZD26078.1 Product:putative tail protein	PI:QWX10325.1 Product:tail length tape measure protein	PI:UAW06838.1 Product:tail length tape-measure protein 1	PI:YP_010054541.1 Product:tail length tape measure protein	PI:YP_010054355.1 Product:tail length tape measure protein	PI:YP_010054429.1 Product:tail length tape measure protein	PI:URY99589.1 Product:tail length tape-measure protein T	PI:UVX29517.1 Product:tail length tape measure protein	PI:UVX30188.1 Product:tail length tape measure protein	PI:UVX30235.1 Product:tail length tape measure protein	PI:UVX30627.1 Product:tail length tape measure protein	PI:UVX31601.1 Product:tail length tape measure protein	PI:UVX31715.1 Product:tail length tape measure protein
PI:UZN98681.1 Product:minor tail protein	PI:QYW02922.1 Product:minor tail protein	PI:YP_008126232.1 Product:hypothetical protein	PI:YP_009614658.1 Product:hypothetical protein	PI:UIW10225.1 Product:hypothetical protein	PI:QZD26079.1 Product:minor tail protein	PI:QWX10326.1 Product:hypothetical protein	PI:UAW06837.1 Product:hypothetical protein	PI:YP_010054542.1 Product:hypothetical protein	PI:YP_010054356.1 Product:hypothetical protein	PI:YP_010054428.1 Product:hypothetical protein	PI:URY99590.1 Product:hypothetical protein	PI:UVX29515.1 Product:virion structural protein	PI:UVX30187.1 Product:virion structural protein	PI:UVX30238.1 Product:virion structural protein	PI:UVX30629.1 Product:virion structural protein	PI:UVX31603.1 Product:virion structural protein	PI:UVX31716.1 Product:virion structural protein
PI:UZN98682.1 Product:hypothetical protein	PI:QYW02923.1 Product:hypothetical protein	PI:YP_008126231.1 Product:minor tail protein	PI:YP_009614659.1 Product:minor tail protein	PI:UIW10227.1 Product:hypothetical protein	PI:QZD26080.1 Product:hypothetical protein	PI:QWX10327.1 Product:hypothetical protein	PI:UAW06836.1 Product:hypothetical protein	PI:YP_010054543.1 Product:minor tail protein	PI:YP_010054357.1 Product:minor tail protein	PI:YP_010054427.1 Product:minor tail protein	PI:URY99591.1 Product:hypothetical protein	PI:UVX29514.1 Product:minor tail protein	PI:UVX30186.1 Product:minor tail protein	PI:UVX30239.1 Product:minor tail protein	PI:UVX30630.1 Product:minor tail protein	PI:UVX31604.1 Product:minor tail protein	PI:UVX31717.1 Product:minor tail protein
PI:UZN98684.1 Product:putative tail protein	PI:QYW02925.1 Product:putative tail protein	PI:YP_008126229.1 Product:tail protein	PI:YP_009614661.1 Product:tail protein	PI:UIW10198.1 Product:hypothetical protein	PI:QZD26082.1 Product:tail protein	PI:QWX10329.1 Product:hypothetical protein	PI:UAW06834.1 Product:hypothetical protein	PI:YP_010054545.1 Product:tail protein	PI:YP_010054358.1 Product:tail protein	PI:YP_010054426.1 Product:tail protein	PI:URY99593.1 Product:hypothetical protein	PI:UVX29512.1 Product:tail protein	PI:UVX30184.1 Product:tail protein	PI:UVX30241.1 Product:tail protein	PI:UVX30632.1 Product:tail protein	PI:UVX31606.1 Product:tail protein	PI:UVX31719.1 Product:tail protein

Table S3. Pan-genome analysis of ZCKP2 and closely related phages (S_G ≥0.6)

The number of homologs in each column is: 31

Klebsiella phage ZCKP2<br>OP481875	Klebsiella phage ZCKP8<br>MZ440881	Klebsiella phage 6991<br>OL362277	Klebsiella phage BUCT610<br>MZ318367	Vibrio phage pYD38-A<br>NC_021534	Aeromonas phage pIS4-A<br>NC_042037	Klebsiella phage VLCpiS13a<br>ON602738	Klebsiella phage vB_KpnS_MK54<br>MW119258	Klebsiella phage VLCpiS13f<br>ON602730	Klebsiella phage VLCpiS13e<br>ON602759	Klebsiella virus KpV2811<br>NC_054653	Klebsiella phage vB_KpnS_ZX4<br>NC_054654	Klebsiella phage BUCT541<br>MZ836210	Flyfo siphovirus Tbat1_6<br>OL617041
PI:UZN98670.1<br>Product:major coat protein	PI:QYW02909.1<br>Product:major coat protein	PI:URY99566.1<br>Product:coat protein	PI:QWX10301.1<br>Product:protein of unknown function DUF2184	PI:YP_008126177.1<br>Product:virion structural protein	PI:YP_009614637.1<br>Product:virion structural protein	PI:UVX30215.1<br>Product:virion structural protein	PI:QZD26058.1<br>Product:major capsid protein	PI:UVX29537.1<br>Product:virion structural protein	PI:UVX31658.1<br>Product:virion structural protein	PI:YP_010054454.1<br>Product:virion structural protein	PI:YP_010054518.1<br>Product:virion structural protein	PI:UAW06863.1<br>Product:coat protein	PI:UIW10206.1<br>Product:protein of unknown function DUF6260
PI:UZN98650.1<br>Product:large terminase subunit	PI:QYW02886.1<br>Product:large terminase subunit	PI:URY99536.1<br>Product:terminase large subunit	PI:QWX10352.1<br>Product:large terminase subunit	PI:YP_008126204.1<br>Product:terminase large subunit	PI:YP_009614610.1<br>Product:terminase large subunit	PI:UVX30263.1<br>Product:terminase large subunit	PI:QZD26107.1<br>Product:large terminase subunit	PI:UVX29570.1<br>Product:terminase large subunit	PI:UVX31628.1<br>Product:terminase large subunit	PI:YP_010054485.1<br>Product:terminase large subunit	PI:YP_010054565.1<br>Product:terminase large subunit	PI:UAW06891.1<br>Product:large terminase	PI:UIW10204.1<br>Product:terminase large subunit
PI:UZN98643.1<br>Product:hypothetical protein	PI:QYW02877.1<br>Product:hypothetical protein	PI:URY99606.1<br>Product:hypothetical protein	PI:QWX10344.1<br>Product:hypothetical protein	PI:YP_008126213.1<br>Product:hypothetical protein	PI:YP_009614601.1<br>Product:hypothetical protein	PI:UVX30255.1<br>Product:hypothetical protein	PI:QZD26098.1<br>Product:hypothetical protein	PI:UVX29578.1<br>Product:hypothetical protein	PI:UVX31619.1<br>Product:hypothetical protein	PI:YP_010054492.1<br>Product:hypothetical protein	PI:YP_010054559.1<br>Product:hypothetical protein	PI:UAW06819.1<br>Product:hypothetical protein	PI:UIW10248.1<br>Product:hypothetical protein
PI:UZN98645.1<br>Product:hypothetical protein	PI:QYW02878.1<br>Product:hypothetical protein	PI:URY99607.1<br>Product:hypothetical protein	PI:QWX10345.1<br>Product:hypothetical protein	PI:YP_008126212.1<br>Product:hypothetical protein	PI:YP_009614602.1<br>Product:hypothetical protein	PI:UVX30256.1<br>Product:hypothetical protein	PI:QZD26099.1<br>Product:hypothetical protein	PI:UVX29577.1<br>Product:hypothetical protein	PI:UVX31620.1<br>Product:hypothetical protein	PI:YP_010054491.1<br>Product:hypothetical protein	PI:YP_010054560.1<br>Product:hypothetical protein	PI:UAW06818.1<br>Product:hypothetical protein	PI:UIW10233.1<br>Product:hypothetical protein
PI:UZN98649.1<br>Product:hypothetical protein	PI:QYW02885.1<br>Product:hypothetical protein	PI:URY99535.1<br>Product:hypothetical protein	PI:QWX10350.1<br>Product:hypothetical protein	PI:YP_008126205.1<br>Product:hypothetical protein	PI:YP_009614609.1<br>Product:hypothetical protein	PI:UVX30262.1<br>Product:hypothetical protein	PI:QZD26106.1<br>Product:hypothetical protein	PI:UVX29571.1<br>Product:hypothetical protein	PI:UVX31626.1<br>Product:hypothetical protein	PI:YP_010054486.1<br>Product:hypothetical protein	PI:YP_010054563.1<br>Product:hypothetical protein	PI:UAW06815.1<br>Product:hypothetical protein	PI:UIW10230.1<br>Product:hypothetical protein
PI:UZN98688.1<br>Product:hypothetical protein	PI:QYW02928.1<br>Product:hypothetical protein	PI:URY99538.1<br>Product:hypothetical protein	PI:QWX10271.1<br>Product:hypothetical protein	PI:YP_008126203.1<br>Product:hypothetical protein	PI:YP_009614611.1<br>Product:hypothetical protein	PI:UVX30264.1<br>Product:hypothetical protein	PI:QZD26108.1<br>Product:hypothetical protein	PI:UVX29568.1<br>Product:hypothetical protein	PI:UVX31630.1<br>Product:hypothetical protein	PI:YP_010054484.1<br>Product:hypothetical protein	PI:YP_010054494.1<br>Product:hypothetical protein	PI:UAW06890.1<br>Product:hypothetical protein	PI:UIW10242.1<br>Product:hypothetical protein
PI:UZN98651.1<br>Product:hypothetical protein	PI:QYW02887.1<br>Product:hypothetical protein	PI:URY99539.1<br>Product:hypothetical protein	PI:QWX10272.1<br>Product:hypothetical protein	PI:YP_008126202.1<br>Product:hypothetical protein	PI:YP_009614612.1<br>Product:hypothetical protein	PI:UVX30265.1<br>Product:hypothetical protein	PI:QZD26109.1<br>Product:hypothetical protein	PI:UVX29567.1<br>Product:hypothetical protein	PI:UVX31631.1<br>Product:hypothetical protein	PI:YP_010054483.1<br>Product:hypothetical protein	PI:YP_010054495.1<br>Product:hypothetical protein	PI:UAW06889.1<br>Product:hypothetical protein	PI:UIW10258.1<br>Product:hypothetical protein
PI:UZN98654.1<br>Product:portal (connector) protein	PI:QYW02893.1<br>Product:portal (connector) protein	PI:URY99544.1<br>Product:portal (connector) protein	PI:QWX10279.1<br>Product:portal protein	PI:YP_008126197.1<br>Product:portal protein	PI:YP_009614617.1<br>Product:portal protein	PI:UVX30272.1<br>Product:portal protein	PI:QZD26115.1<br>Product:portal protein	PI:UVX29561.1<br>Product:portal protein	PI:UVX31635.1<br>Product:portal protein	PI:YP_010054478.1<br>Product:portal protein	PI:YP_010054497.1<br>Product:portal protein	PI:UAW06885.1<br>Product:hypothetical protein	PI:UIW10203.1<br>Product:protein of unknown function DUF1073
PI:UZN98656.1<br>Product:head decoration protein	PI:QYW02895.1<br>Product:head decoration protein	PI:URY99545.1<br>Product:hypothetical protein	PI:QWX10280.1<br>Product:hypothetical protein	PI:YP_008126196.1<br>Product:head morphogenesis	PI:YP_009614618.1<br>Product:head morphogenesis	PI:UVX30273.1<br>Product:head morphogenesis	PI:QZD26116.1<br>Product:minor capsid protein	PI:UVX29560.1<br>Product:head morphogenesis	PI:UVX31636.1<br>Product:head morphogenesis	PI:YP_010054477.1<br>Product:head morphogenesis	PI:YP_010054498.1<br>Product:head morphogenesis	PI:UAW06884.1<br>Product:hypothetical protein	PI:UIW10207.1<br>Product:hypothetical protein
PI:UZN98690.1<br>Product:hypothetical protein	PI:QYW02932.1<br>Product:hypothetical protein	PI:URY99549.1<br>Product:hypothetical protein	PI:QWX10284.1<br>Product:hypothetical protein	PI:YP_008126193.1<br>Product:hypothetical protein	PI:YP_009614621.1<br>Product:hypothetical protein	PI:UVX30276.1<br>Product:hypothetical protein	PI:QZD26120.1<br>Product:hypothetical protein	PI:UVX29557.1<br>Product:hypothetical protein	PI:UVX31640.1<br>Product:hypothetical protein	PI:YP_010054472.1<br>Product:hypothetical protein	PI:YP_010054502.1<br>Product:hypothetical protein	PI:UAW06881.1<br>Product:hypothetical protein	PI:UIW10244.1<br>Product:hypothetical protein
PI:UZN98657.1<br>Product:lysozyme	PI:QYW02896.1<br>Product:lysozyme	PI:URY99550.1<br>Product:lysozyme R	PI:QWX10285.1<br>Product:lysozyme	PI:YP_008126192.1<br>Product:endolysin	PI:YP_009614622.1<br>Product:endolysin	PI:UVX30277.1<br>Product:endolysin	PI:QZD26122.1<br>Product:lysozyme	PI:UVX29556.1<br>Product:endolysin	PI:UVX31641.1<br>Product:endolysin	PI:YP_010054471.1<br>Product:endolysin	PI:YP_010054503.1<br>Product:endolysin	PI:UAW06880.1<br>Product:lysozyme	PI:UIW10247.1<br>Product:hypothetical protein
PI:UZN98693.1<br>Product:hypothetical protein	PI:QYW02936.1<br>Product:hypothetical protein	PI:URY99554.1<br>Product:hypothetical protein	PI:QWX10290.1<br>Product:hypothetical protein	PI:YP_008126189.1<br>Product:hypothetical protein	PI:YP_009614625.1<br>Product:hypothetical protein	PI:UVX30283.1<br>Product:hypothetical protein	PI:QZD26047.1<br>Product:hypothetical protein	PI:UVX29549.1<br>Product:hypothetical protein	PI:UVX31647.1<br>Product:hypothetical protein	PI:YP_010054466.1<br>Product:hypothetical protein	PI:YP_010054507.1<br>Product:hypothetical protein	PI:UAW06874.1<br>Product:hypothetical protein	PI:UIW10220.1<br>Product:hypothetical protein
PI:UZN98694.1<br>Product:holin	PI:QYW02937.1<br>Product:holin	PI:URY99556.1<br>Product:hypothetical protein	PI:QWX10291.1<br>Product:hypothetical protein	PI:YP_008126187.1<br>Product:hypothetical protein	PI:YP_009614627.1<br>Product:hypothetical protein	PI:UVX30285.1<br>Product:hypothetical protein	PI:QZD26049.1<br>Product:hypothetical protein	PI:UVX29547.1<br>Product:hypothetical protein	PI:UVX31649.1<br>Product:hypothetical protein	PI:YP_010054465.1<br>Product:hypothetical protein	PI:YP_010054508.1<br>Product:hypothetical protein	PI:UAW06873.1<br>Product:hypothetical protein	PI:UIW10267.1<br>Product:hypothetical protein
PI:UZN98660.1<br>Product:hypothetical protein	PI:QYW02899.1<br>Product:hypothetical protein	PI:URY99557.1<br>Product:hypothetical protein	PI:QWX10292.1<br>Product:hypothetical protein	PI:YP_008126186.1<br>Product:hypothetical protein	PI:YP_009614628.1<br>Product:hypothetical protein	PI:UVX30286.1<br>Product:hypothetical protein	PI:QZD26050.1<br>Product:hypothetical protein	PI:UVX29546.1<br>Product:hypothetical protein	PI:UVX31650.1<br>Product:hypothetical protein	PI:YP_010054463.1<br>Product:hypothetical protein	PI:YP_010054509.1<br>Product:hypothetical protein	PI:UAW06872.1<br>Product:hypothetical protein	PI:UIW10263.1<br>Product:hypothetical protein
PI:UZN98664.1<br>Product:hypothetical protein	PI:QYW02903.1<br>Product:hypothetical protein	PI:URY99561.1<br>Product:hypothetical protein	PI:QWX10296.1<br>Product:hypothetical protein	PI:YP_008126182.1<br>Product:MarR family transcrition regulator	PI:YP_009614632.1<br>Product:MarR family transcrition regulator	PI:UVX30210.1<br>Product:MarR family transcrition regulator	PI:QZD26053.1<br>Product:hypothetical protein	PI:UVX29542.1<br>Product:MarR family transcrition regulator	PI:UVX31653.1<br>Product:MarR family transcrition regulator	PI:YP_010054459.1<br>Product:MarR family transcrition regulator	PI:YP_010054513.1<br>Product:MarR family transcrition regulator	PI:UAW06868.1<br>Product:transcription factor	PI:UIW10226.1<br>Product:hypothetical protein
PI:UZN98666.1<br>Product:hypothetical protein	PI:QYW02905.1<br>Product:hypothetical protein	PI:URY99563.1<br>Product:hypothetical protein	PI:QWX10298.1<br>Product:hypothetical protein	PI:YP_008126180.1<br>Product:hypothetical protein	PI:YP_009614634.1<br>Product:hypothetical protein	PI:UVX30212.1<br>Product:hypothetical protein	PI:QZD26055.1<br>Product:hypothetical protein	PI:UVX29540.1<br>Product:hypothetical protein	PI:UVX31655.1<br>Product:hypothetical protein	PI:YP_010054457.1<br>Product:hypothetical protein	PI:YP_010054515.1<br>Product:hypothetical protein	PI:UAW06866.1<br>Product:hypothetical protein	PI:UIW10237.1<br>Product:hypothetical protein
PI:UZN98668.1<br>Product:hypothetical protein	PI:QYW02907.1<br>Product:hypothetical protein	PI:URY99564.1<br>Product:coat protein	PI:QWX10299.1<br>Product:coat protein	PI:YP_008126179.1<br>Product:hypothetical protein	PI:YP_009614635.1<br>Product:hypothetical protein	PI:UVX30213.1<br>Product:hypothetical protein	PI:QZD26056.1<br>Product:prohead core protein serine protease	PI:UVX29539.1<br>Product:hypothetical protein	PI:UVX31656.1<br>Product:hypothetical protein	PI:YP_010054456.1<br>Product:hypothetical protein	PI:YP_010054516.1<br>Product:coat protein	PI:UAW06865.1<br>Product:hypothetical protein	PI:UIW10205.1<br>Product:hypothetical protein
PI:UZN98697.1<br>Product:hypothetical protein	PI:QYW02940.1<br>Product:hypothetical protein	PI:URY99571.1<br>Product:hypothetical protein	PI:QWX10306.1<br>Product:hypothetical protein	PI:YP_008126171.1<br>Product:hypothetical protein	PI:YP_009614643.1<br>Product:hypothetical protein	PI:UVX30220.1<br>Product:hypothetical protein	PI:QZD26062.1<br>Product:hypothetical protein	PI:UVX29534.1<br>Product:hypothetical protein	PI:UVX31662.1<br>Product:hypothetical protein	PI:YP_010054450.1<br>Product:hypothetical protein	PI:YP_010054521.1<br>Product:hypothetical protein	PI:UAW06859.1<br>Product:hypothetical protein	PI:UIW10276.1<br>Product:hypothetical protein
PI:UZN98699.1<br>Product:DNA binding protein	PI:QYW02942.1<br>Product:putative transcriptional regulator	PI:URY99573.1<br>Product:hypothetical protein	PI:QWX10308.1<br>Product:hypothetical protein	PI:YP_008126170.1<br>Product:ParB-like partition protein	PI:YP_009614644.1<br>Product:ParB-like partition protein	PI:UVX30222.1<br>Product:hypothetical protein	PI:QZD26064.1<br>Product:putative transcriptional regulator	PI:UVX29532.1<br>Product:ParB-like partition protein	PI:UVX31664.1<br>Product:ParB-like partition protein	PI:YP_010054447.1<br>Product:ParB-like partition protein	PI:YP_010054524.1<br>Product:ParB-like partition protein	PI:UAW06856.1<br>Product:hypothetical protein	PI:UIW10212.1<br>Product:KorB domain protein
PI:UZN98673.1<br>Product:hypothetical protein	PI:QYW02912.1<br>Product:hypothetical protein	PI:URY99576.1<br>Product:hypothetical protein	PI:QWX10311.1<br>Product:hypothetical protein	PI:YP_008126168.1<br>Product:membrane protein	PI:YP_009614646.1<br>Product:membrane protein	PI:UVX30223.1<br>Product:membrane protein	PI:QZD26068.1<br>Product:holin	PI:UVX29528.1<br>Product:membrane protein	PI:UVX31588.1<br>Product:membrane protein	PI:YP_010054444.1<br>Product:membrane protein	PI:YP_010054527.1<br>Product:membrane protein	PI:UAW06853.1<br>Product:hypothetical protein	PI:UIW10245.1<br>Product:hypothetical protein
PI:UZN98674.1<br>Product:hypothetical protein	PI:QYW02913.1<br>Product:hypothetical protein	PI:URY99577.1<br>Product:hypothetical protein	PI:QWX10312.1<br>Product:hypothetical protein	PI:YP_008126167.1<br>Product:hypothetical protein	PI:YP_009614647.1<br>Product:hypothetical protein	PI:UVX30224.1<br>Product:hypothetical protein	PI:QZD26069.1<br>Product:head to tail adaptor	PI:UVX29527.1<br>Product:hypothetical protein	PI:UVX31589.1<br>Product:hypothetical protein	PI:YP_010054443.1<br>Product:hypothetical protein	PI:YP_010054528.1<br>Product:hypothetical protein	PI:UAW06852.1<br>Product:hypothetical protein	PI:UIW10235.1<br>Product:hypothetical protein
PI:UZN98675.1<br>Product:hypothetical protein	PI:QYW02915.1<br>Product:hypothetical protein	PI:URY99579.1<br>Product:hypothetical protein	PI:QWX10313.1<br>Product:hypothetical protein	PI:YP_008126166.1<br>Product:hypothetical protein	PI:YP_009614648.1<br>Product:hypothetical protein	PI:UVX30226.1<br>Product:hypothetical protein	PI:QZD26070.1<br>Product:minor capsid protein	PI:UVX29526.1<br>Product:hypothetical protein	PI:UVX31591.1<br>Product:hypothetical protein	PI:YP_010054441.1<br>Product:hypothetical protein	PI:YP_010054530.1<br>Product:hypothetical protein	PI:UAW06850.1<br>Product:minor capsid protein	PI:UIW10236.1<br>Product:hypothetical protein
PI:UZN98676.1<br>Product:hypothetical protein	PI:QYW02916.1<br>Product:hypothetical protein	PI:URY99580.1<br>Product:hypothetical protein	PI:QWX10314.1<br>Product:hypothetical protein	PI:YP_008126165.1<br>Product:HK97 gp10 family protein	PI:YP_009614649.1<br>Product:HK97 gp10 family protein	PI:UVX30227.1<br>Product:tail completion or Neck1 protein	PI:QZD26071.1<br>Product:minor capsid protein	PI:UVX29525.1<br>Product:hypothetical protein	PI:UVX31592.1<br>Product:hypothetical protein	PI:YP_010054440.1<br>Product:HK97 gp10 family protein	PI:YP_010054531.1<br>Product:HK97 gp10 family protein	PI:UAW06849.1<br>Product:hypothetical protein	PI:UIW10232.1<br>Product:hypothetical protein

PI:UZN98677.1<br>Product:hypothetical protein	PI:QYW02917.1<br>Product:hypothetical protein	PI:URY99581.1<br>Product:hypothetical protein	PI:QWX10315.1<br>Product:hypothetical protein	PI:YP_008126240.1<br>Product:hypothetical protein	PI:YP_009614650.1<br>Product:hypothetical protein	PI:UVX30228.1<br>Product:hypothetical protein	PI:QZD26072.1<br>Product:tail completion protein	PI:UVX29524.1<br>Product:hypothetical protein	PI:UVX31593.1<br>Product:hypothetical protein	PI:YP_010054439.1<br>Product:hypothetical protein	PI:YP_010054532.1<br>Product:hypothetical protein	PI:UAW06848.1<br>Product:hypothetical protein	PI:UIW10234.1<br>Product:hypothetical protein
PI:UZN98678.1<br>Product:major tail subunit	PI:QYW02919.1<br>Product:major tail subunit	PI:URY99583.1<br>Product:hypothetical protein	PI:QWX10318.1<br>Product:hypothetical protein	PI:YP_008126238.1<br>Product:major tail protein	PI:YP_009614652.1<br>Product:major tail protein	PI:UVX30229.1<br>Product:major tail protein	PI:QZD26073.1<br>Product:major tail subunit	PI:UVX29523.1<br>Product:major tail protein	PI:UVX31595.1<br>Product:major tail protein	PI:YP_010054437.1<br>Product:major tail protein	PI:YP_010054534.1<br>Product:major tail protein	PI:UAW06845.1<br>Product:major tail protein	PI:UIW10213.1<br>Product:Immunoglobulin domain protein
PI:UZN98679.1<br>Product:hypothetical protein	PI:QYW02920.1<br>Product:hypothetical protein	PI:URY99588.1<br>Product:hypothetical protein	PI:QWX10324.1<br>Product:hypothetical protein	PI:YP_008126234.1<br>Product:DUF6246 family protein	PI:YP_009614656.1<br>Product:DUF6246 family protein	PI:UVX30234.1<br>Product:hypothetical protein	PI:QZD26077.1<br>Product:hypothetical protein	PI:UVX29518.1<br>Product:hypothetical protein	PI:UVX31600.1<br>Product:hypothetical protein	PI:YP_010054430.1<br>Product:DUF6246 family protein	PI:YP_010054540.1<br>Product:DUF6246 family protein	PI:UAW06839.1<br>Product:hypothetical protein	PI:UIW10216.1<br>Product:protein of unknown function DUF6246
PI:UZN98680.1<br>Product:tail length tape measure protein	PI:QYW02921.1<br>Product:tail length tape measure protein	PI:URY99589.1<br>Product:tail length tape-measure protein T	PI:QWX10325.1<br>Product:tail length tape measure protein	PI:YP_008126233.1<br>Product:tail length tape measure protein	PI:YP_009614657.1<br>Product:tail length tape measure protein	PI:UVX30235.1<br>Product:tail length tape measure protein	PI:QZD26078.1<br>Product:putative tail protein	PI:UVX29517.1<br>Product:tail length tape measure protein	PI:UVX31601.1<br>Product:tail length tape measure protein	PI:YP_010054429.1<br>Product:tail length tape measure protein	PI:YP_010054541.1<br>Product:tail length tape measure protein	PI:UAW06838.1<br>Product:tail length tape-measure protein 1	PI:UIW10199.1<br>Product:tape measure protein
PI:UZN98681.1<br>Product:minor tail protein	PI:QYW02922.1<br>Product:minor tail protein	PI:URY99590.1<br>Product:hypothetical protein	PI:QWX10326.1<br>Product:hypothetical protein	PI:YP_008126232.1<br>Product:hypothetical protein	PI:YP_009614658.1<br>Product:hypothetical protein	PI:UVX30238.1<br>Product:virion structural protein	PI:QZD26079.1<br>Product:minor tail protein	PI:UVX29515.1<br>Product:virion structural protein	PI:UVX31603.1<br>Product:virion structural protein	PI:YP_010054428.1<br>Product:hypothetical protein	PI:YP_010054542.1<br>Product:hypothetical protein	PI:UAW06837.1<br>Product:hypothetical protein	PI:UIW10225.1<br>Product:hypothetical protein
PI:UZN98682.1<br>Product:hypothetical protein	PI:QYW02923.1<br>Product:hypothetical protein	PI:URY99591.1<br>Product:hypothetical protein	PI:QWX10327.1<br>Product:hypothetical protein	PI:YP_008126231.1<br>Product:minor tail protein	PI:YP_009614659.1<br>Product:minor tail protein	PI:UVX30239.1<br>Product:minor tail protein	PI:QZD26080.1<br>Product:hypothetical protein	PI:UVX29514.1<br>Product:minor tail protein	PI:UVX31604.1<br>Product:minor tail protein	PI:YP_010054427.1<br>Product:minor tail protein	PI:YP_010054543.1<br>Product:minor tail protein	PI:UAW06836.1<br>Product:hypothetical protein	PI:UIW10227.1<br>Product:hypothetical protein
PI:UZN98684.1<br>Product:putative tail protein	PI:QYW02925.1<br>Product:putative tail protein	PI:URY99593.1<br>Product:hypothetical protein	PI:QWX10329.1<br>Product:hypothetical protein	PI:YP_008126229.1<br>Product:tail protein	PI:YP_009614661.1<br>Product:tail protein	PI:UVX30241.1<br>Product:tail protein	PI:QZD26082.1<br>Product:tail protein	PI:UVX29512.1<br>Product:tail protein	PI:UVX31606.1<br>Product:tail protein	PI:YP_010054426.1<br>Product:tail protein	PI:YP_010054545.1<br>Product:tail protein	PI:UAW06834.1<br>Product:hypothetical protein	PI:UIW10198.1<br>Product:hypothetical protein
PI:UZN98704.1<br>Product:single-stranded DNA-binding protein	PI:QYW02950.1<br>Product:single-stranded DNA-binding protein	PI:URY99597.1<br>Product:hypothetical protein	PI:QWX10332.1<br>Product:single-stranded DNA-binding protein	PI:YP_008126226.1<br>Product:single strand DNA binding protein	PI:YP_009614664.1<br>Product:single strand DNA binding protein	PI:UVX30243.1<br>Product:single strand DNA binding protein	PI:QZD26084.1<br>Product:single-stranded DNA-binding protein	PI:UVX29591.1<br>Product:single strand DNA binding protein	PI:UVX31608.1<br>Product:single strand DNA binding protein	PI:YP_010054424.1<br>Product:single strand DNA binding protein	PI:YP_010054547.1<br>Product:single strand DNA binding protein	PI:UAW06832.1<br>Product:single strand binding protein	PI:UIW10224.1<br>Product:single-strand binding protein family protein

Table S4. Pan-genome analysis of ZCKP2 and closely related phages (SG ≥0.7)

The number of homologs in each column is: 54

**Klebsiella phage ZCKP2**<br><br>**OP481875**	**Klebsiella phage ZCKP8**<br><br>**MZ440881**	**Klebsiella phage 6991**<br><br>**OL362277**
PI:UZN98670.1<br><br>Product:major coat protein	PI:QYW02909.1<br><br>Product:major coat protein	PI:URY99566.1<br><br>Product:coat protein
PI:UZN98650.1<br><br>Product:large terminase subunit	PI:QYW02886.1<br><br>Product:large terminase subunit	PI:URY99536.1<br><br>Product:terminase large subunit
PI:UZN98704.1<br><br>Product:single-stranded DNA-binding protein	PI:QYW02950.1<br><br>Product:single-stranded DNA-binding protein	PI:URY99597.1<br><br>Product:hypothetical protein
PI:UZN98642.1<br><br>Product:hypothetical protein	PI:QYW02876.1<br><br>Product:hypothetical protein	PI:URY99605.1<br><br>Product:hypothetical protein
PI:UZN98643.1<br><br>Product:hypothetical protein	PI:QYW02877.1<br><br>Product:hypothetical protein	PI:URY99606.1<br><br>Product:hypothetical protein
PI:UZN98645.1<br><br>Product:hypothetical protein	PI:QYW02878.1<br><br>Product:hypothetical protein	PI:URY99607.1<br><br>Product:hypothetical protein
PI:UZN98644.1<br><br>Product:minor tail protein	PI:QYW02879.1<br><br>Product:hypothetical protein	PI:URY99608.1<br><br>Product:hypothetical protein
PI:UZN98646.1<br><br>Product:EaA-like protein	PI:QYW02880.1<br><br>Product:hypothetical protein	PI:URY99609.1<br><br>Product:hypothetical protein
PI:UZN98647.1<br><br>Product:hypothetical protein	PI:QYW02882.1<br><br>Product:hypothetical protein	PI:URY99611.1<br><br>Product:EaA protein
PI:UZN98648.1<br><br>Product:hypothetical protein	PI:QYW02884.1<br><br>Product:hypothetical protein	PI:URY99614.1<br><br>Product:hypothetical protein
PI:UZN98649.1<br><br>Product:hypothetical protein	PI:QYW02885.1<br><br>Product:hypothetical protein	PI:URY99535.1<br><br>Product:hypothetical protein
PI:UZN98688.1<br><br>Product:hypothetical protein	PI:QYW02928.1<br><br>Product:hypothetical protein	PI:URY99538.1<br><br>Product:hypothetical protein
PI:UZN98651.1<br><br>Product:hypothetical protein	PI:QYW02887.1<br><br>Product:hypothetical protein	PI:URY99539.1<br><br>Product:hypothetical protein
PI:UZN98654.1<br><br>Product:portal (connector) protein	PI:QYW02893.1<br><br>Product:portal (connector) protein	PI:URY99544.1<br><br>Product:portal (connector) protein

PI:UZN98656.1<br><br>Product:head decoration protein	PI:QYW02895.1<br><br>Product:head decoration protein	PI:URY99545.1<br><br>Product:hypothetical protein
PI:UZN98689.1<br><br>Product:hypothetical protein	PI:QYW02930.1<br><br>Product:hypothetical protein	PI:URY99547.1<br><br>Product:hypothetical protein
PI:UZN98690.1<br><br>Product:hypothetical protein	PI:QYW02932.1<br><br>Product:hypothetical protein	PI:URY99549.1<br><br>Product:hypothetical protein
PI:UZN98657.1<br><br>Product:lysozyme	PI:QYW02896.1<br><br>Product:lysozyme	PI:URY99550.1<br><br>Product:lysozyme R
PI:UZN98658.1<br><br>Product:holin protein	PI:QYW02897.1<br><br>Product:putative holin	PI:URY99551.1<br><br>Product:hypothetical protein
PI:UZN98659.1<br><br>Product:tail protein	PI:QYW02898.1<br><br>Product:tail protein	PI:URY99552.1<br><br>Product:lysin
PI:UZN98693.1<br><br>Product:hypothetical protein	PI:QYW02936.1<br><br>Product:hypothetical protein	PI:URY99554.1<br><br>Product:hypothetical protein
PI:UZN98694.1<br><br>Product:holin	PI:QYW02937.1<br><br>Product:holin	PI:URY99556.1<br><br>Product:hypothetical protein
PI:UZN98660.1<br><br>Product:hypothetical protein	PI:QYW02899.1<br><br>Product:hypothetical protein	PI:URY99557.1<br><br>Product:hypothetical protein
PI:UZN98661.1<br><br>Product:hypothetical protein	PI:QYW02900.1<br><br>Product:hypothetical protein	PI:URY99558.1<br><br>Product:hypothetical protein
PI:UZN98662.1<br><br>Product:hypothetical protein	PI:QYW02901.1<br><br>Product:hypothetical protein	PI:URY99559.1<br><br>Product:hypothetical protein
PI:UZN98663.1<br><br>Product:zinc-binding domain of primase-helicase	PI:QYW02902.1<br><br>Product:zinc-binding domain of primase-helicase	PI:URY99560.1<br><br>Product:hypothetical protein
PI:UZN98664.1<br><br>Product:hypothetical protein	PI:QYW02903.1<br><br>Product:hypothetical protein	PI:URY99561.1<br><br>Product:hypothetical protein
PI:UZN98665.1<br><br>Product:hypothetical protein	PI:QYW02904.1<br><br>Product:hypothetical protein	PI:URY99562.1<br><br>Product:hypothetical protein
PI:UZN98666.1<br><br>Product:hypothetical protein	PI:QYW02905.1<br><br>Product:hypothetical protein	PI:URY99563.1<br><br>Product:hypothetical protein
PI:UZN98668.1<br><br>Product:hypothetical protein	PI:QYW02907.1<br><br>Product:hypothetical protein	PI:URY99564.1<br><br>Product:coat protein
PI:UZN98669.1<br><br>Product:capsid decoration protein	PI:QYW02908.1<br><br>Product:capsid decoration protein	PI:URY99565.1<br><br>Product:hypothetical protein
PI:UZN98671.1<br><br>Product:hypothetical protein	PI:QYW02910.1<br><br>Product:hypothetical protein	PI:URY99567.1<br><br>Product:hypothetical protein

PI:UZN98672.1<br><br>Product:hypothetical protein	PI:QYW02911.1<br><br>Product:hypothetical protein	PI:URY99568.1<br><br>Product:hypothetical protein
PI:UZN98695.1<br><br>Product:hypothetical protein	PI:QYW02938.1<br><br>Product:hypothetical protein	PI:URY99569.1<br><br>Product:hypothetical protein
PI:UZN98696.1<br><br>Product:DNA polymerase III beta subunit	PI:QYW02939.1<br><br>Product:DNA polymerase III beta subunit	PI:URY99570.1<br><br>Product:DNA polymerase III beta subunit
PI:UZN98697.1<br><br>Product:hypothetical protein	PI:QYW02940.1<br><br>Product:hypothetical protein	PI:URY99571.1<br><br>Product:hypothetical protein
PI:UZN98698.1<br><br>Product:hypothetical protein	PI:QYW02941.1<br><br>Product:hypothetical protein	PI:URY99572.1<br><br>Product:hypothetical protein
PI:UZN98699.1<br><br>Product:DNA binding protein	PI:QYW02942.1<br><br>Product:putative transcriptional regulator	PI:URY99573.1<br><br>Product:hypothetical protein
PI:UZN98700.1<br><br>Product:hypothetical protein	PI:QYW02944.1<br><br>Product:hypothetical protein	PI:URY99575.1<br><br>Product:hypothetical protein
PI:UZN98673.1<br><br>Product:hypothetical protein	PI:QYW02912.1<br><br>Product:hypothetical protein	PI:URY99576.1<br><br>Product:hypothetical protein
PI:UZN98674.1<br><br>Product:hypothetical protein	PI:QYW02913.1<br><br>Product:hypothetical protein	PI:URY99577.1<br><br>Product:hypothetical protein
PI:UZN98675.1<br><br>Product:hypothetical protein	PI:QYW02915.1<br><br>Product:hypothetical protein	PI:URY99579.1<br><br>Product:hypothetical protein
PI:UZN98676.1<br><br>Product:hypothetical protein	PI:QYW02916.1<br><br>Product:hypothetical protein	PI:URY99580.1<br><br>Product:hypothetical protein
PI:UZN98677.1<br><br>Product:hypothetical protein	PI:QYW02917.1<br><br>Product:hypothetical protein	PI:URY99581.1<br><br>Product:hypothetical protein
PI:UZN98678.1<br><br>Product:major tail subunit	PI:QYW02919.1<br><br>Product:major tail subunit	PI:URY99583.1<br><br>Product:hypothetical protein
PI:UZN98701.1<br><br>Product:hypothetical protein	PI:QYW02946.1<br><br>Product:hypothetical protein	PI:URY99584.1<br><br>Product:hypothetical protein
PI:UZN98702.1<br><br>Product:hypothetical protein	PI:QYW02947.1<br><br>Product:hypothetical protein	PI:URY99585.1<br><br>Product:hypothetical protein
PI:UZN98679.1<br><br>Product:hypothetical protein	PI:QYW02920.1<br><br>Product:hypothetical protein	PI:URY99588.1<br><br>Product:hypothetical protein
PI:UZN98680.1<br><br>Product:tail length tape measure protein	PI:QYW02921.1<br><br>Product:tail length tape measure protein	PI:URY99589.1<br><br>Product:tail length tape-measure protein T

PI:UZN98681.1<br><br>Product:minor tail protein	PI:QYW02922.1<br><br>Product:minor tail protein	PI:URY99590.1<br><br>Product:hypothetical protein
PI:UZN98682.1<br><br>Product:hypothetical protein	PI:QYW02923.1<br><br>Product:hypothetical protein	PI:URY99591.1<br><br>Product:hypothetical protein
PI:UZN98683.1<br><br>Product:tail assembly protein	PI:QYW02924.1<br><br>Product:tail assembly protein	PI:URY99592.1<br><br>Product:hypothetical protein
PI:UZN98684.1<br><br>Product:putative tail protein	PI:QYW02925.1<br><br>Product:putative tail protein	PI:URY99593.1<br><br>Product:hypothetical protein
PI:UZN98685.1<br><br>Product:putative tail fiber protein	PI:QYW02926.1<br><br>Product:tail fiber protein	PI:URY99594.1<br><br>Product:hypothetical protein

Table S5. Pan-genome analysis of ZCKP2 and closely related phages (SG ≥0.9)

The number of homologs in each column is: 69

**Klebsiella phage ZCKP2**<br><br>**OP481875**	**Klebsiella phage ZCKP8**<br><br>**MZ440881**
PI:UZN98670.1<br><br>Product:major coat protein	PI:QYW02909.1<br><br>Product:major coat protein
PI:UZN98650.1<br><br>Product:large terminase subunit	PI:QYW02886.1<br><br>Product:large terminase subunit
PI:UZN98704.1<br><br>Product:single-stranded DNA-binding protein	PI:QYW02950.1<br><br>Product:single-stranded DNA-binding protein
PI:UZN98696.1<br><br>Product:DNA polymerase III beta subunit	PI:QYW02939.1<br><br>Product:DNA polymerase III beta subunit
PI:UZN98642.1<br><br>Product:hypothetical protein	PI:QYW02876.1<br><br>Product:hypothetical protein
PI:UZN98643.1<br><br>Product:hypothetical protein	PI:QYW02877.1<br><br>Product:hypothetical protein
PI:UZN98645.1<br><br>Product:hypothetical protein	PI:QYW02878.1<br><br>Product:hypothetical protein
PI:UZN98644.1<br><br>Product:minor tail protein	PI:QYW02879.1<br><br>Product:hypothetical protein
PI:UZN98646.1<br><br>Product:EaA-like protein	PI:QYW02880.1<br><br>Product:hypothetical protein
PI:UZN98647.1<br><br>Product:hypothetical protein	PI:QYW02882.1<br><br>Product:hypothetical protein
PI:UZN98648.1<br><br>Product:hypothetical protein	PI:QYW02884.1<br><br>Product:hypothetical protein
PI:UZN98649.1<br><br>Product:hypothetical protein	PI:QYW02885.1<br><br>Product:hypothetical protein
PI:UZN98688.1<br><br>Product:hypothetical protein	PI:QYW02928.1<br><br>Product:hypothetical protein
PI:UZN98651.1<br><br>Product:hypothetical protein	PI:QYW02887.1<br><br>Product:hypothetical protein
PI:UZN98652.1<br><br>Product:hypothetical protein	PI:QYW02890.1<br><br>Product:hypothetical protein

PI:UZN98653.1<br><br>Product:hypothetical protein	PI:QYW02892.1<br><br>Product:hypothetical protein
PI:UZN98654.1<br><br>Product:portal (connector) protein	PI:QYW02893.1<br><br>Product:portal (connector) protein
PI:UZN98655.1<br><br>Product:hypothetical protein	PI:QYW02894.1<br><br>Product:hypothetical protein
PI:UZN98656.1<br><br>Product:head decoration protein	PI:QYW02895.1<br><br>Product:head decoration protein
PI:UZN98689.1<br><br>Product:hypothetical protein	PI:QYW02930.1<br><br>Product:hypothetical protein
PI:UZN98690.1<br><br>Product:hypothetical protein	PI:QYW02932.1<br><br>Product:hypothetical protein
PI:UZN98657.1<br><br>Product:lysozyme	PI:QYW02896.1<br><br>Product:lysozyme
PI:UZN98658.1<br><br>Product:holin protein	PI:QYW02897.1<br><br>Product:putative holin
PI:UZN98659.1<br><br>Product:tail protein	PI:QYW02898.1<br><br>Product:tail protein
PI:UZN98691.1<br><br>Product:hypothetical protein	PI:QYW02933.1<br><br>Product:hypothetical protein
PI:UZN98692.1<br><br>Product:hypothetical protein	PI:QYW02935.1<br><br>Product:hypothetical protein
PI:UZN98693.1<br><br>Product:hypothetical protein	PI:QYW02936.1<br><br>Product:hypothetical protein
PI:UZN98694.1<br><br>Product:holin	PI:QYW02937.1<br><br>Product:holin
PI:UZN98660.1<br><br>Product:hypothetical protein	PI:QYW02899.1<br><br>Product:hypothetical protein
PI:UZN98661.1<br><br>Product:hypothetical protein	PI:QYW02900.1<br><br>Product:hypothetical protein
PI:UZN98662.1<br><br>Product:hypothetical protein	PI:QYW02901.1<br><br>Product:hypothetical protein
PI:UZN98663.1<br><br>Product:zinc-binding domain of primase-helicase	PI:QYW02902.1<br><br>Product:zinc-binding domain of primase-helicase
PI:UZN98664.1<br><br>Product:hypothetical protein	PI:QYW02903.1<br><br>Product:hypothetical protein

PI:UZN98665.1<br><br>Product:hypothetical protein	PI:QYW02904.1<br><br>Product:hypothetical protein
PI:UZN98666.1<br><br>Product:hypothetical protein	PI:QYW02905.1<br><br>Product:hypothetical protein
PI:UZN98667.1<br><br>Product:hypothetical protein	PI:QYW02906.1<br><br>Product:hypothetical protein
PI:UZN98668.1<br><br>Product:hypothetical protein	PI:QYW02907.1<br><br>Product:hypothetical protein
PI:UZN98669.1<br><br>Product:capsid decoration protein	PI:QYW02908.1<br><br>Product:capsid decoration protein
PI:UZN98671.1<br><br>Product:hypothetical protein	PI:QYW02910.1<br><br>Product:hypothetical protein
PI:UZN98672.1<br><br>Product:hypothetical protein	PI:QYW02911.1<br><br>Product:hypothetical protein
PI:UZN98695.1<br><br>Product:hypothetical protein	PI:QYW02938.1<br><br>Product:hypothetical protein
PI:UZN98697.1<br><br>Product:hypothetical protein	PI:QYW02940.1<br><br>Product:hypothetical protein
PI:UZN98698.1<br><br>Product:hypothetical protein	PI:QYW02941.1<br><br>Product:hypothetical protein
PI:UZN98699.1<br><br>Product:DNA binding protein	PI:QYW02942.1<br><br>Product:putative transcriptional regulator
PI:UZN98700.1<br><br>Product:hypothetical protein	PI:QYW02944.1<br><br>Product:hypothetical protein
PI:UZN98673.1<br><br>Product:hypothetical protein	PI:QYW02912.1<br><br>Product:hypothetical protein
PI:UZN98674.1<br><br>Product:hypothetical protein	PI:QYW02913.1<br><br>Product:hypothetical protein
PI:UZN98675.1<br><br>Product:hypothetical protein	PI:QYW02915.1<br><br>Product:hypothetical protein
PI:UZN98676.1<br><br>Product:hypothetical protein	PI:QYW02916.1<br><br>Product:hypothetical protein
PI:UZN98677.1<br><br>Product:hypothetical protein	PI:QYW02917.1<br><br>Product:hypothetical protein
PI:UZN98678.1<br><br>Product:major tail subunit	PI:QYW02919.1<br><br>Product:major tail subunit

PI:UZN98701.1<br><br>Product:hypothetical protein	PI:QYW02946.1<br><br>Product:hypothetical protein
PI:UZN98702.1<br><br>Product:hypothetical protein	PI:QYW02947.1<br><br>Product:hypothetical protein
PI:UZN98703.1<br><br>Product:HNH endonuclease	PI:QYW02948.1<br><br>Product:HNH-endonuclease
PI:UZN98679.1<br><br>Product:hypothetical protein	PI:QYW02920.1<br><br>Product:hypothetical protein
PI:UZN98680.1<br><br>Product:tail length tape measure protein	PI:QYW02921.1<br><br>Product:tail length tape measure protein
PI:UZN98681.1<br><br>Product:minor tail protein	PI:QYW02922.1<br><br>Product:minor tail protein
PI:UZN98682.1<br><br>Product:hypothetical protein	
PI:UZN98683.1<br><br>Product:tail assembly protein	PI:QYW02924.1<br><br>Product:tail assembly protein
PI:UZN98684.1<br><br>Product:putative tail protein	PI:QYW02925.1<br><br>Product:putative tail protein
PI:UZN98685.1<br><br>Product:putative tail fiber protein	PI:QYW02926.1<br><br>Product:tail fiber protein
PI:UZN98705.1<br><br>Product:recombinase	PI:QYW02951.1<br><br>Product:hypothetical protein
PI:UZN98706.1<br><br>Product:exonuclease	PI:QYW02952.1<br><br>Product:exonuclease
PI:UZN98707.1<br><br>Product:hypothetical protein	PI:QYW02953.1<br><br>Product:hypothetical protein
PI:UZN98708.1<br><br>Product:hypothetical protein	PI:QYW02954.1<br><br>Product:hypothetical protein
PI:UZN98709.1<br><br>Product:HNH endonuclease	PI:QYW02956.1<br><br>Product:putative endonuclease protein
PI:UZN98686.1<br><br>Product:DNA primase	PI:QYW02927.1<br><br>Product:DNA primase
PI:UZN98711.1<br><br>Product:hypothetical protein	PI:QYW02958.1<br><br>Product:hypothetical protein
PI:UZN98712.1<br><br>Product:hypothetical protein	PI:QYW02959.1<br><br>Product:hypothetical protein

Table S6. Pan-genome analysis of ZCKP2 and phages of *Drexlerviridae* family (SG ≥0.05)

The number of homologs in each column is:7

**Klebsiella phage ZCKP2**<br><br>**OP481875**	**Klebsiella phage MezzoGao**<br><br>**NC_047850**	**Klebsiella phage KpKT21phi1**<br><br>**NC_048143**	**Klebsiella phage vB_KpnS_15-38_KLPPOU149**<br><br>**NC_049842**	**Klebsiella phage GML-KpCol1**<br><br>**NC_047907**
PI:UZN98704.1<br><br>Product:single-stranded DNA-binding protein	PI:YP_009792118.1<br><br>Product:single-stranded DNA binding protein	PI:YP_009818761.1<br><br>Product:hypothetical protein	PI:YP_009903159.1<br><br>Product:hypothetical protein	PI:YP_009796908.1<br><br>Product:hypothetical protein
PI:UZN98701.1<br><br>Product:hypothetical protein	PI:YP_009792141.1<br><br>Product:DNA methyltransferase	PI:YP_009818815.1<br><br>Product:DNA methyltransferase	PI:YP_009903104.1<br><br>Product:DNA methyltransferase	PI:YP_009796964.1<br><br>Product:DNA methyltransferase
PI:UZN98705.1<br><br>Product:recombinase	PI:YP_009792119.1<br><br>Product:Erf-like ssDNA annealing protein	PI:YP_009818760.1<br><br>Product:Erf-like ssDNA annealing protein	PI:YP_009903160.1<br><br>Product:Erf-like ssDNA annealing protein	PI:YP_009796907.1<br><br>Product:Erf-like ssDNA annealing protein
PI:UZN98706.1<br><br>Product:exonuclease	PI:YP_009792120.1<br><br>Product:exonuclease VIII	PI:YP_009818759.1<br><br>Product:exonuclease VIII	PI:YP_009903161.1<br><br>Product:exonuclease VIII	PI:YP_009796906.1<br><br>Product:exonuclease VIII
PI:UZN98708.1<br><br>Product:hypothetical protein	PI:YP_009792124.1<br><br>Product:VRR-NUC domain-containing protein	PI:YP_009818831.1<br><br>Product:VRR-NUC domain-containing protein	PI:YP_009903088.1<br><br>Product:VRR-NUC domain-containing protein	PI:YP_009796902.1<br><br>Product:VRR-NUC domain-containing protein
PI:UZN98686.1<br><br>Product:DNA primase	PI:YP_009792121.1<br><br>Product:DNA primase	PI:YP_009818758.1<br><br>Product:DNA primase	PI:YP_009903162.1<br><br>Product:DNA primase	PI:YP_009796905.1<br><br>Product:DNA primase
PI:UZN98711.1<br><br>Product:hypothetical protein	PI:YP_009792137.1<br><br>Product:DNA helicase	PI:YP_009818819.1<br><br>Product:DNA helicase	PI:YP_009903100.1<br><br>Product:DNA helicase	PI:YP_009796968.1<br><br>Product:DNA helicase

Table S7. Number of predicted orthologues genes between phage ZCKP2 and other phages

Classification	Accession#	Name	ViPTree *SG*	Orthologs by CoreGenes 0.5						
				24	31	54	69	7	1	0
Unclassified Siphoviruses	MZ440881	Klebsiella phage_ZCKP8	0.9277							
	OL362277	Klebsiella phage_6991	0.7025							
	MZ318367	Klebsiella phage_BUCT610	0.6553							
	NC_021534	Vibrio phage pYD38-A	0.6547							
	NC_042037	Aeromonas phage pIS4-A	0.6537							
	ON602738	Klebsiella phage_VLCpiS13a	0.652							
	MW119258	Klebsiella phage__vB_KpnS_MK54	0.6262							
	ON602730	Klebsiella phage_VLCpiS13f	0.6236							
	ON602759	Klebsiella phage_VLCpiS13e	0.6022							
	NC_054653	Klebsiella virus KpV2811	0.6018							
	NC_054654	Klebsiella phage vB_KpnS_ZX4	0.6013							
	MZ836210	Klebsiella phage_BUCT541	0.5972							
	OL617041	Siphovirus_Tbat1_6	0.5934							
	ON602737	Klebsiella phage_VLCpiS13c	0.5684							
	ON602760	Klebsiella phage_VLCpiS13d	0.5336							
	ON602744	Klebsiella phage_VLCpiS13b	0.5028							
	NC_054652	Klebsiella phage YX3973	0.4959							
Drexlerviridae	NC_047850	Klebsiella phage MezzoGao	0.0558							
	NC_048143	Klebsiella phage KpKT21phi1	0.0522							
	NC_049842	Klebsiella phage vB_KpnS_15-38_KLPPOU149	0.0514							
	NC_047907	Klebsiella phage GML-KpCol1	0.0519							
Myov.	NC_049462	Klebsiella phage Magnus	Outgroups							
	NC_047901	Klebsiella phage Menlow								
Podov.	NC_047748	Klebsiella phage phiBO1E								
	NC_047771	Klebsiella phage vB_KpnP_KpV763								
